# Reciprocal associations between parental feeding practices and child eating behaviours from toddlerhood to early childhood: bivariate latent change analysis in the Gemini cohort

**DOI:** 10.1111/jcpp.13819

**Published:** 2023-05-15

**Authors:** Alice R. Kininmonth, Moritz Herle, Emma Haycraft, Clare Farrow, Kristiane Tommerup, Helen Croker, Abigail Pickard, Katie Edwards, Jacqueline Blissett, Clare Llewellyn

**Affiliations:** ^1^ Research Department of Behavioural Science and Health, Institute of Epidemiology and Health Care University College London London UK; ^2^ Social, Genetic & Developmental Psychiatry Centre, Institute of Psychiatry, Psychology & Neuroscience King's College London London UK; ^3^ School of Sport, Exercise and Health Sciences Loughborough University Loughborough UK; ^4^ School of Psychology, Institute of Health and Neurodevelopment Aston University Birmingham UK; ^5^ World Cancer Research Fund International London UK

**Keywords:** Reciprocal, parental feeding practices, children, eating behaviour

## Abstract

**Background:**

Parental feeding practices (PFPs) are a key component of a child's food environment. Parent–child feeding relationships are hypothesised to be bidirectional; however, to date, few large prospective studies have examined this, instead focussing on unidirectional relationships. As such, the direction of relationships between PFPs and children's eating behaviours remains unclear.

**Methods:**

Data were from Gemini, a population‐based sample of children born in England and Wales in 2007. Children's eating behaviours and PFPs were measured at 15/16 months and 5 years using validated psychometric measures (*n* = 1,858 children). Bivariate Latent Change Score Modelling was used to examine the nature of relationships between PFPs and children's eating behaviours at 15/16 months and 5 years. Models were adjusted to account for clustering of twins within families and for sex of the child, socioeconomic status, gestational age and age of the child at measurement time points.

**Results:**

A reciprocal relationship was observed between instrumental feeding and emotional overeating, with greater instrumental feeding predicting greater increases in emotional overeating (*β* = .09; 0.03–0.15; *p* = .004) and vice versa (*β* = .09; 0.03–0.15; *p* = .005). Reciprocity was also observed between encouragement to eat nutritious foods and children's enjoyment of food, with greater encouragement predicting greater increases in enjoyment of food (*β* = .08; 0.02–0.13; *p* = .006) and vice versa (*β* = .07; 0.02–0.11; *p* = .003). Parent–child associations and child–parent associations were also observed.

**Conclusion:**

These findings are consistent with the hypothesis that certain feeding practices are used as a ‘natural’ response to a child expressing a greater interest in and enthusiasm for food, but at the same time, such practices impact the development of eating behaviours by nurturing and encouraging the expression of higher emotional overeating and greater enjoyment of food in preschool years. The findings provide important insights into the PFPs and eating behaviour traits that could be targeted as part of a tailored feeding intervention to support parents of children during the preschool formative years.

## Introduction

Parental feeding practices (PFPs) are a key component of a child's food environment (Savage, Fisher, & Birch, 2007). Parents are the nutritional ‘gatekeepers’ of their children's food, especially during the preschool years and, as such, PFPs are often the focus of many childhood obesity prevention strategies (Gomes, Pereira, Roberto, Boraska, & Barros, 2021). Evidence from cross‐sectional and prospective studies suggest that PFPs may nurture the development of children's eating behaviours and weight outcomes (Carnell, Benson, Driggin, & Kolbe, 2014; Carnell & Wardle, 2007b; Musher‐Eizenman & Holub, 2007; Russell et al., 2018).

Nonresponsive feeding practices that are controlling or coercive are hypothesised to undermine a child's ability to self‐regulate their food intake in response to their internal hunger and satiety cues. Using food to control or manipulate a child's behaviour (such as rewarding good behaviour with food—also termed instrumental feeding) or emotion (termed emotional feeding) is hypothesised to teach a child to exalt food beyond its nutritional purpose, and value it as a reward or a coping strategy for soothing negative emotions. Using food as a reward or to soothe emotions has been shown to positively predict subsequent food responsiveness (Berge et al., 2020) and emotional overeating (Steinsbekk, Belsky, & Wichstrom, 2016). Excessively restricting a child's access to their favourite palatable foods is also hypothesised to enhance their responsiveness to such foods, by virtue of the ‘forbidden fruit effect’ (put simply, children want what they are not allowed to have). In support of this hypothesis, experimental studies have shown an increase in a child's desire to obtain and consume food that has been restricted (Fisher & Birch, 1999a, 1999b). Pressuring a child to eat is also hypothesised to compromise the child's ability to regulate their food intake, by overriding their internal feelings of satiety and teaching the child to respond more to external than internal cues to eat (e.g. to consume everything on the plate, regardless of hunger level) (Birch, Birch, Marlin, & Kramer, 1982; Birch, McPheee, Shoba, Steinberg, & Krehbiel, 1987; Newman & Taylor, 1992). Contrary to this hypothesis, two large prospective studies in school‐aged children (4–6 years; *n* = 4,845; 4–7 years; *n* = 3,698) have shown that greater pressure to eat at age 4 predicted greater fussiness around food (Jansen et al., 2017) and slower speed of eating (Costa, Severo, & Oliveira, 2021), which may be due to learned aversion from the upset and anxiety caused by pressuring feeding practices. These types of nonresponsive feeding practices may contribute towards the development of childhood obesity or eating disorders in adolescence, by nurturing obesogenic eating behaviours and/or an unhealthy relationship with food, and hence are promising intervention targets to help parents to support the healthy growth of their children.

Much of the research to date has been unidirectional, focussing on relationships from parent to child, rather than the reverse. Models of child development (Black & Aboud, 2011) suggest that relationships are likely to be bidirectional in nature; in the child‐feeding domain, the way a parent feeds their child may influence their child's eating behaviours and, in turn, parents also develop their feeding practices in response to their child's emerging weight status or unique eating style (Steinsbekk et al., 2016). To date, few longitudinal studies have examined the reciprocity of parent–child feeding relationships in large representative samples. The findings have been inconsistent, and studies have focussed on a limited number of nonresponsive PFPs and child eating behaviour traits. Findings from an Australian study conducted in a small sample of mother–child dyads (*n* = 207; followed from 3.7 to 5 years) identified a bi‐directional relationship between a child's food fussiness and PFPs, with higher fussiness predicting more nonresponsive feeding while nonresponsive feeding also predicted higher food fussiness (Mallan et al., 2018). In another example, a Norwegian cohort (*n* = 797; followed from 4 to 10 years) observed a reciprocal relationship between child emotional eating and parental emotional feeding (Steinsbekk, Barker, Llewellyn, Fildes, & Wichstrøm, 2018) and found that greater parental use of instrumental feeding predicted greater increases in emotional overeating and food responsiveness (Steinsbekk et al., 2016) but did not observe the reverse relationship from child to parent.

As PFPs are potentially modifiable, understanding the nature of the relationships between PFPs and children's eating behaviours is particularly important. Currently, knowledge is hampered by the lack of longitudinal research in large representative cohorts examining the directionality of associations between PFPs and children's food approach traits, and to date, there have been no studies in the preschool formative years. Therefore, the purpose of this study was to examine the directionality of the relationships between a comprehensive range of PFPs and children's eating behaviour traits from 15/16 months to 5 years.

## Methods

### Sample

Participants were from the Gemini study, a longitudinal birth cohort of families with twins born in England and Wales between March and December 2007. 2,402 families with monozygotic (identical) and dizygotic (non‐identical) twins (*n* = 4804) consented to take part and completed baseline questionnaires when their children were a mean (±*SD*) of 8.2 (±2.2) months old. The recruitment of the sample has been described in detail elsewhere (van Jaarsveld, Johnson, Llewellyn, & Wardle, 2010). Data used in this study are from baseline, 15/16 months, and 5 years. Of the 2,402 families who completed the baseline questionnaire, 1,931 families (80.4%) completed the 15/16 months questionnaire, and 1,087 families (45.3%) completed the 5 years questionnaire. This study sample comprised 929 families (1,858 children; 955 [51.4%] female) with complete data on all variables included in the analysis. The twins' primary caregiver provided written informed consent for their family to participate in the study. Ethical approval was granted by the University College London Committee for the Ethics of non‐National Health Service Human Research. The Gemini dataset was used as it is one of the most comprehensive and largest UK‐based longitudinal birth cohorts with repeated measures of weight, height, a wide range of eating behaviours, PFPs and sociodemographic characteristics, from early life, that was available to the authors which allowed the research question to be addressed.

### Measures

#### Parental feeding practices

Eight PFPs were reported by the primary caregiver when their children were 15/16 months and 5 years old (Table S1; Birch et al., 2001; Musher‐Eizenman & Holub, 2007; Ogden, Reynolds, & Smith, 2006; Wardle, Sanderson, Guthrie, Rapoport, & Plomin, 2002). The eight scales included three nonresponsive (Instrumental feeding, Emotional feeding, Pressure to eat) and five responsive PFPs (Covert restriction, Control over meals/snacks, Monitoring, Encouragement to eat nutritious foods, Modelling). ‘Emotional feeding’ measures caregivers' use of food to manage or control a child's negative emotions (5 items; ‘I give my child something to eat to make him/her feel better when s/he is feeling upset’; *15/16 months: α = .85, 5 years: α = .80*). ‘Pressure to eat’ measures caregivers' attempts to coerce the child to eat more (5 items; e.g. ‘My child should always eat all of the food I give him/her’; *15/16 months: α = .65, 5 years: α = .63*). ‘Covert restriction’ measures the extent to which parents restrict their child's access to foods, supposedly without their child knowing (4 items; e.g. ‘I avoid buying unhealthy foods and bringing them into the house’; *15/16 months: α = .69, 5 years: α = .71*). ‘Instrumental feeding’ measures caregivers' use of food as a contingency for healthy food consumption or good behaviour (4 items; e.g. *‘I use puddings as a bribe to get my child to eat his/her main course’; 15/16 months: α = .50, 5 years: α = .68*). The ‘Parent Control’ scale examines the extent to which caregivers exert control over what and when their child eats meals and snacks (5 items; *‘I decide how many snacks my child should have’; 15/16 months: α = .58, 5 years: α = .65*). ‘Encouragement to eat’ assesses caregivers use of positive reinforcement to encourage their child to eat food, particularly healthy foods (5 items; e.g. *‘I encourage my child to eat a wide variety of foods’; 15/16 months: α = .59, 5 years: α = .63*). ‘Monitoring’ assesses the extent to which caregivers keep track of their child's high fat/sugary food consumption while in their own or others' care (3 items; e.g. *‘I keep track of the high fat foods that my child eats’; 15/16 months: α = .72, 5 years: α = .73*). ‘Modelling’ assesses the extent to which caregivers model healthy eating to their children (4 items; e.g. *‘I model healthy eating for my child by eating healthy foods myself’; 15/16 months: α = .80, 5 years: α = .80*). Items were rated using a 5‐point Likert scale from *never* (1) to *always* (5). A mean score was calculated for each of the scales for each twin if responses were available for most items within a scale (2/3 for monitoring, 3/4 for modelling and covert restriction, and 3/5 items for remaining scales).

#### Child eating behaviour

Child eating behaviour was assessed at 5 years using the Child Eating Behaviour Questionnaire (CEBQ) and at 15/16 months using the CEBQ‐T (toddler version of the CEBQ). The CEBQ is a validated parent‐reported psychometric measure of eight eating behaviour traits (seven eating behaviour traits and one drinking behaviour trait), which consists of 35 items, rated using a 5‐point Likert scale (1 = *Never* to 5 = *Always*; Carnell & Wardle, 2007a; Wardle, Guthrie, Sanderson, & Rapoport, 2001). All eating behaviour scales were included, except for, emotional undereating which could not be included as it was removed from the CEBQ‐T (toddler version) because mothers reported during piloting of the measure that their toddlers did not engage in these behaviours (Herle, Fildes, van Jaarsveld, Rijsdijk, & Llewellyn, 2016). Food Responsiveness (FR) measures a child's drive to eat in response to external food cues (5 items e.g. *‘Given the choice, my child would eat most of the time’; 15/16 months: α = .76, 5 years: α = .81*). Enjoyment of Food (EF) assesses a child's subjective pleasure from eating (4 items, e.g. *‘My child loves food’; 15/16 months: α = .85, 5 years: α = .86*). Emotional Overeating (EOE; 4 items, e.g. *‘My child eats more when worried’; 15/16 months: α = .82, 5 years: α = .77*) assesses the extent to which a child eats more in response to emotional stressors. Satiety Responsiveness (SR) measures a child's sensitivity to internal cues of ‘fullness’ (5 items e.g. *‘My child gets full up easily’; 15/16 months: α = .75, 5 years: α = .76*). Slowness in Eating (SE) refers to the speed of meal consumption (4 items, e.g. *‘My child eats slowly’; 15/16 months: α = .66, 5 years: α = .79*). Finally, Food Fussiness (FF) examines a child's pickiness about the flavour and texture of foods they are willing to eat (6 items; e.g. ‘My child refuses new foods at first’; *15/16 months: α = .87, 5 years: α = .91*). A mean score was calculated for each subscale for participants who had completed the majority of items for that scale (3/4 for EOE, EF, SE, 3/5 for FR, SR, 4/6 for FF).

#### Covariates

Caregivers reported the sex, date of birth, birth weight (kg) and gestational age of their twins in the baseline questionnaires and provided information about indicators of socioeconomic status (SES), including maternal educational qualification; current occupation (both parents); annual household income; postcode; home ownership status; number of cars and bedrooms at home. Principal component analysis was used to create the SES composite score, which incorporated the seven SES indicators. Scores ranged from 1.30 to 6.96, with higher scores reflecting higher SES. Full details are described elsewhere (Kininmonth, Smith, Llewellyn, & Fildes, 2020).

### Statistical analyses

**Figure 1:**
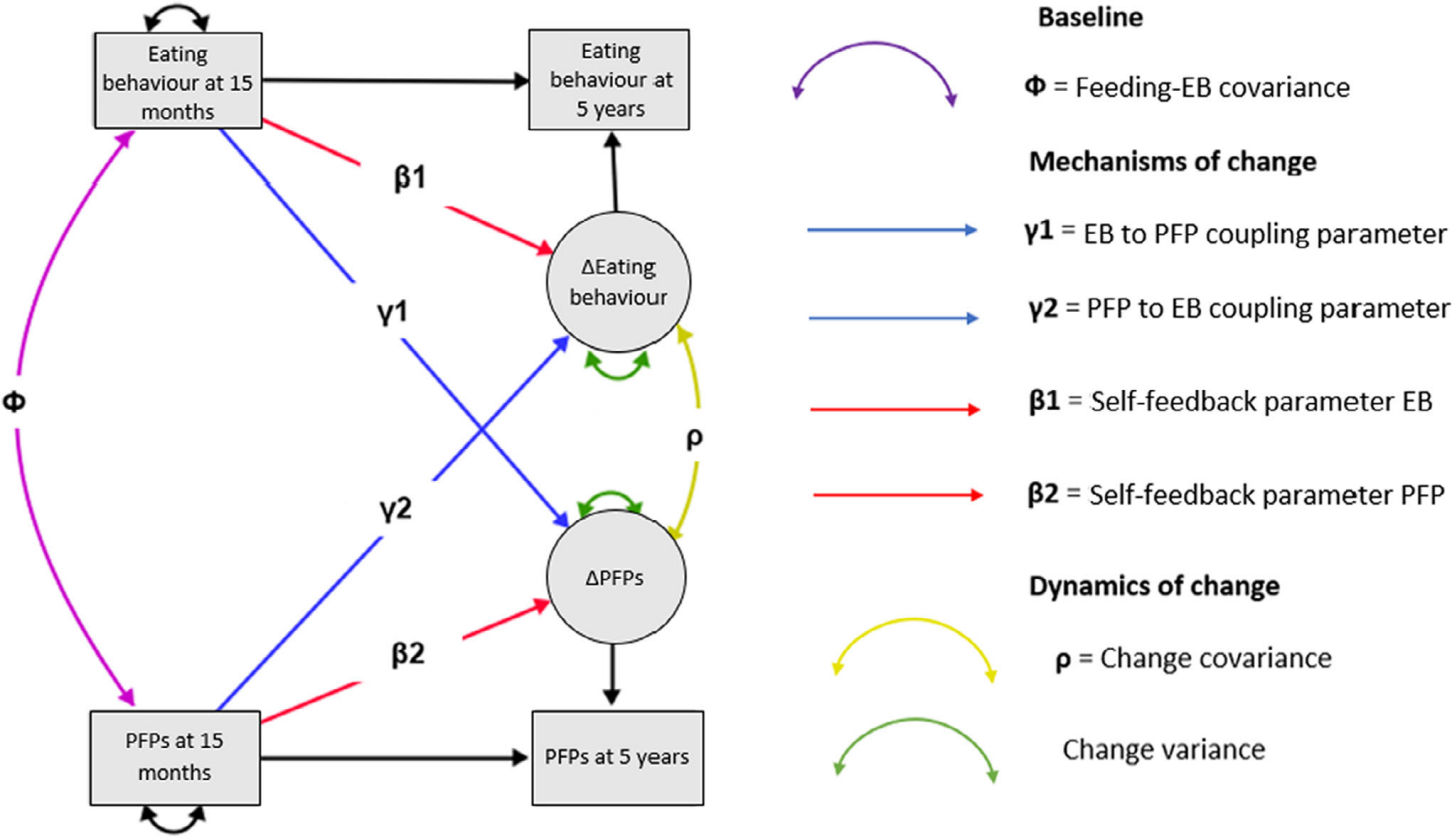
Schematic to describe the Bivariate Latent Change Score Model (LCSM). The bivariate LCSM estimates four parent feeding‐eating behaviour relations of interest, these are shown in purple, blue and yellow. Firstly, feeding‐eating behaviour covariance at baseline (*Ф*), shown in purple. Secondly, eating behaviour to parent feeding coupling, where a child's eating behaviour at 15/16 months (T1) predicts the rate or degree of change in parental feeding, shown in blue (*γ*1). Thirdly, parent feeding to child eating behaviour coupling, where parental feeding scores at 15/16 months (T1) predict the rate or degree of change in child eating behaviour, shown in blue (*γ*2). Finally, there is an estimation of correlated change, shown in yellow (*ρ*), reflecting the degree to which PFPs and eating behaviour changes co‐occur after taking into account the coupling parameters [Color figure can be viewed at wileyonlinelibrary.com]

Bivariate latent change score models (LCSM) were computed using Stata version 17 (StataCorp., College Station, TX, USA) to test dynamic change between PFPs and children's eating behaviour traits from toddlerhood to early childhood. Changes in PFPs and children's eating behaviours were modelled as latent change scores to identify contributions of baseline measurements on respective outcomes. LCSM are powerful analytic methods as they allow for investigation of the concept of cross‐domain coupling, that captures the extent to which change in one domain (e.g. parental feeding) is a function of starting level in the other domain (e.g. child eating behaviour), and vice versa (Kievit et al., 2018). LCSM go above and beyond usual longitudinal models as discussed elsewhere (Kievit et al., 2018) and have been applied to understand dynamic changes in various psychological domains, such as peer relationships (Rappaport et al., 2021), exercise drive and eating disorder symptoms (Chapa, Kite, Forbush, Tregarthen, & Argue, 2020). However, this method has not been applied to examine the bi‐directional longitudinal associations between PFPs and child eating behaviour. The bivariate LCSM estimates four parent feeding‐eating behaviours trait relations of interest; these are shown in purple, blue and yellow in Figure 1. Separate SEM models were fitted for each PFP‐child eating behaviours trait pair.

Models were fitted using maximum likelihood estimation, adjusting for clustering of twins within families, using the clustered sandwich estimator in Stata. Models were also adjusted for covariates (SES, child sex, child age at measurement timepoints, gestational age), with paths drawn from the covariates to the latent change scores. Model fit indices were calculated, with cut‐offs in parentheses indicating acceptable to good fit: Comparative Fit Index (CFI ≥ 0.90), Root Mean Square Error of Approximation (RMSEA ≤ 0.10) and Standardised Root Mean Square Residuals (SRMR ≤ 0.08; Hooper, Coughlan, & Mullen, 2008). Model fit indices were acceptable to good for all models included in the analysis and could therefore be interpreted further. To allow for full transparency and to maximise comparability by other researchers, all results are presented in full, with 95% confidence intervals (CI) and *p*‐values. This approach has been used as it allows researchers to apply adjustment for multiple testing in the way that they feel most appropriate. Due to word count restrictions, only significant (*α* < .01) prospective paths will be discussed in the results section. The more conservative alpha level of .01 was used due to the large sample size. Standardised beta coefficients are presented throughout the manuscript; therefore, Cohen's guidelines for classification of effect sizes were used to interpret the size of effect (Cohen, 2013). For a coefficient *β*, effect sizes <0.29 were classified as small, between 0.30–0.49 were medium and >0.50 classified as large (Cohen, 2013; Nieminen, 2022).

Analyses were repeated using a full information maximum likelihood estimator. This allows for the inclusion of all data points regardless of attrition, under the assumption of missing (completely) at random (MAR/MCAR). Sample size for these models was *N* = 3,787. Characteristics of the sample with missing data due to attrition compared with the sample with complete data are presented in Table S2. Full results are presented in Tables 3, 4, 5 and S3–S7, with full information maximum likelihood results presented in Tables S8–S15 and significant associations are summarised in Table 2.

## Results

The sample analysed included 1,858 twins with complete data on all study variables. The characteristics of the analysis sample are shown in Table 1. Model fit indices for all SEM models interpreted in the results were acceptable to good. For full details of CFI, RMSEA and SRMR, see figure captions for each model, Tables 3, 4, 5 and S3–S7.

**Table 1:** Descriptive statistics for analysis sample with complete data at 15/16 months and 5 years (*n* = 1,858 twins, 929 families)

Sample characteristics	Mean (*SD*) or *N* (%)	
Child sex		
Female	955 (51.4%)	
Gestational age (weeks)^a^	36.26 (2.44)	
Maternal age at twin birth (years)^b^	33.92 (4.59)	
Child age at 15/16 months (months)	15.64 (0.95)	
Child age at 5 years (years)	5.15 (0.13)	
SES composite score^c^	4.64 (1.25)	
Ethnicity		
White‐British	1,668 (89.9)	
White Other	110 (5.9)	
Black, Black British, Caribbean and African	14 (0.8)	
Asian or Asian British	34 (1.8)	
Mixed or multiple ethnic groups	28 (1.5)	
Other ethnic group	4 (0.2)	
**Child eating behaviours**	**15/16 months**	**5 years**
Food responsiveness	2.23 (0.75)	2.36 (0.74)
Emotional overeating	1.63 (0.58)	1.56 (0.51)
Enjoyment of food	4.16 (0.62)	3.89 (0.67)
Satiety responsiveness	2.68 (0.63)	2.86 (0.62)
Slowness in eating	2.47 (0.65)	2.83 (0.77)
**Parental feeding practices**	**15/16 months**	**5 years**
Emotional feeding	2.00 (0.72)	1.70 (0.55)
Pressure to eat	2.21 (0.71)	2.75 (0.66)
Instrumental feeding	1.69 (0.51)	2.33 (0.62)
Covert restriction	3.07 (0.90)	2.99 (0.80)
Control	4.49 (0.45)	4.15 (0.44)
Monitoring	3.86 (0.98)	3.55 (0.91)
Encouragement	4.03 (0.62)	4.14 (0.52)
Modelling	3.40 (0.83)	3.73 (0.70)

^a^ Missing data for 5 families, *n* = 924.

^b^ Missing data for 1 family, *n* = 928.

^c^ SES composite scores ranged from 1.30 to 6.96; lower scores on the composite reflect lower SES.

**Table 2:** Overview of the significant associations between eating behaviour traits and parental feeding practices between 15 months and 5 years

Parental feeding practices	Eating behaviour traits					
	Food responsiveness	Emotional overeating	Enjoyment of food	Satiety responsiveness	Slowness in eating	Food fussiness
Nonresponsive feeding practices						
Instrumental feeding	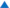	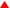 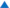				
Emotional feeding	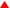	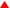				
Pressure to eat	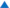	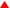			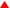	
Responsive feeding practices						
Modelling			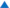			
Encouragement to eat nutritious foods			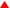 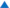			
Control over meals and snacks						
Monitoring of intake			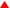			
Covert restriction						

Key: Red arrows in an upward direction indicate that higher levels of the parental feeding practices at 15 months predicted greater increases in the eating behaviour trait from 15 months to 5 years. Blue arrows in an upward direction indicate that higher levels of the eating behaviour trait at 15 months predicted greater increases in the parental feeding practice from 15 months to 5 years.

**Table 3:** Parameter estimates for bivariate latent change model^a^ between instrumental feeding and five child eating behaviour traits

Parameter		Estimate	95% CI		*p*‐Value	Fit statistics^b^
Emotional overeating and instrumental feeding (INS)						CFI = 0.98 RMSEA = 0.05 SRMR = 0.014
Coupling parameter EOE to ∆INS	*γ*1	0.09	0.03	0.15	.007	
Coupling parameter INS to ∆EOE	*γ*2	0.09	0.03	0.15	.004	
Self‐feedback parameter EOE	*β*1	−0.78	−0.72	−0.84	<.001	
Self‐feedback parameter INS	*β*2	−0.58	−0.51	−0.66	<.001	
Covariance EOE and INS at 15/16 months	*Ф*	0.05	0.03	0.07	<.001	
Correlated change	*ρ*	0.07	0.05	0.08	<.001	
Food responsiveness and instrumental feeding (INS)						CFI = 0.994 RMSEA = 0.037 SRMR = 0.010
Coupling parameter FR to ∆INS	*γ*1	**0.12**	**0.07**	**0.17**	**<.001**	
Coupling parameter INS to ∆FR	*γ*2	0.08	−0.00	0.16	.05	
Self‐feedback parameter FR	*β*1	−0.59	−0.53	−0.64	<.001	
Self‐feedback parameter INS	*β*2	−0.59	−0.52	−0.67	<.001	
Covariance between FR and INS at 15/16 months	*Ф*	0.06	0.03	0.08	<.001	
Correlated change	*ρ*	0.08	0.05	0.10	<.001	
Enjoyment of food and instrumental feeding (INS)						CFI = 0.968 RMSEA = 0.074 SRMR = 0.016
Coupling parameter EF to ∆INS	*γ*1	0.01	−0.04	0.07	.624	
Coupling parameter INS to ∆EF	*γ*2	0.00	−0.06	0.07	.965	
Self‐feedback parameter EF	*β*1	−0.52	−0.46	−0.57	<.001	
Self‐feedback parameter INS	*β*2	−0.57	−0.49	−0.64	<.001	
Covariance between EF and INS at 15/16 months	*Ф*	−0.02	−0.00	−0.04	.042	
Correlated change	*ρ*	−0.02	−0.04	0.00	.081	
Satiety responsiveness and instrumental feeding (INS)						CFI = 0.963 RMSEA = 0.079 SRMR = 0.019
Coupling parameter SR to ∆INS	*γ*1	−0.04	−0.10	0.02	.238	
Coupling parameter INS to ∆SR	*γ*2	0.01	−0.05	0.07	.734	
Self‐feedback parameter SR	*β*1	−0.59	−0.54	−0.65	<.001	
Self‐feedback parameter INS	*β*2	−0.56	−0.49	−0.64	<.001	
Covariance between SR and INS at 15/16 months	*Ф*	0.02	0.00	0.04	.033	
Correlated change	*ρ*	0.05	0.03	0.07	<.001	
Slowness in eating and instrumental feeding (INS)						CFI = 0.956 RMSEA = 0.071 SRMR = 0.017
Coupling parameter SE to ∆INS	*γ*1	−0.02	−0.72	0.04	.576	
Coupling parameter INS to ∆SE	*γ*2	0.08	0.00	0.15	.039	
Self‐feedback parameter SE	*β*1	−0.68	−0.62	−0.74	<.001	
Self‐feedback parameter INS	*β*2	−0.57	−0.49	−0.64	<.001	
Covariance between SE and INS at 15/16 months	*Ф*	0.01	−0.01	0.03	.395	
Correlated change	*ρ*	0.05	0.03	0.07	<.001	
Food fussiness and instrumental feeding (INS)						CFI = 0.989 RMSEA = 0.045 SRMR = 0.011
Coupling parameter FF to ∆INS	*γ*1	0.04	−0.01	0.10	.135	
Coupling parameter INS to ∆FF	*γ*2	0.05	−0.03	0.14	.223	
Self‐feedback parameter FF	*β*1	−0.50	−0.44	−0.56	<.001	
Self‐feedback parameter INS	*β*2	−0.58	−0.50	−0.65	<.001	
Covariance between FF and INS at 15/16 months	*Ф*	0.06	0.04	0.08	<.001	
Correlated change	*ρ*	0.07	0.04	0.09	<.001	

*Explanation of the parameters: Coupling parameters (γ1 and γ2)* – reflects the extent to which baseline levels in one domain (e.g. eating behaviour or parent feeding) predicts the rate or degree of change (∆) in the other domain (e.g. parent feeding or eating behaviour). A positive relationship for the *eating behaviour to parent feeding coupling parameter (γ1)* would indicate that higher eating behaviour trait scores at 15/16 months predicted greater increases in the feeding practice from 15/16 months to 5 years. A positive relationship for the *parent feeding to eating behaviour coupling parameter (γ2)* would indicate that higher feeding scores at 15/16 months predicted greater increases in the eating behaviour trait from 15/16 months to 5 years. *Self‐feedback parameters (β1 and β2)* – reflects the extent to which baseline levels in one domain (e.g. eating behaviour or parent feeding) influences change in the same domain (e.g. eating behaviour or parental feeding). The self‐feedback parameter is often negative which reflects regression towards the mean or ceiling effects and should not be overinterpreted. *Covariance at 15/16 months (Φ)* – reflects the covariance between feeding practices and eating behaviour at baseline (15/16 months). *Correlated change (ρ)* – reflects the degree to which PFPs changes and eating behaviour changes co‐occur after taking into account the coupling parameters. A positive relationship for correlated change indicates that over the 4‐year period, greater increases in the eating behaviour trait were also associated with greater increases in the parental feeding practice.

^a^ All models were adjusted for clustering within families and covariates; age of child at measurement (15/16 months and 5 years), SES, gestational age, sex of child. Significant results for the parameters of interest in this study are shown in bold.

^b^ Model fit indices were calculated, with cut‐offs in parentheses indicating acceptable to good fit: Comparative Fit Index (CFI ≥ 0.90), Root Mean Square Error of Approximation (RMSEA ≤ 0.10) and Standardised Root Mean Square Residuals (SRMR ≤ 0.08).

**Table 4:** Parameter estimates for bivariate latent change model^a^ between encouragement and five child eating behaviour traits

Parameter		Estimate	95% CI		*p*‐Value	Fit statistics^b^
Emotional overeating and encouragement (ENC)						CFI = 0.936 SRMR = 0.023 RMSEA = 0.106
Coupling parameter EOE to ∆ENC	*γ*1	−0.05	−0.11	0.00	.068	
Coupling parameter ENC to ∆EOE	*γ*2	−0.004	−0.05	0.05	.886	
Self‐feedback parameter EOE	*β*1	−0.77	−0.71	−0.82	<.001	
Self‐feedback parameter ENC	*β*2	−0.58	−0.53	−0.63	<.001	
Covariance between EOE and ENC at 15/16 months	*Ф*	−0.002	−0.02	0.02	.871	
Correlated change	*ρ*	−0.01	−0.02	0.01	.248	
Food responsiveness and encouragement (ENC)						RMSEA = 0.094 CFI = 0.963 SRMR = 0.020
Coupling parameter FR to ∆ENC	*γ*1	−0.00	−0.04	0.04	.993	
Coupling parameter ENC to ∆FR	*γ*2	−0.003	−0.06	0.06	.933	
Self‐feedback parameter FR	*β*1	−0.58	−0.52	−0.63	<.001	
Self‐feedback parameter ENC	*β*2	−0.58	−0.53	−0.63	<.001	
Covariance between FR and ENC at 15/16 months	*Ф*	0.002	−0.03	0.03	.875	
Correlated change	*ρ*	−0.01	−0.03	0.01	.439	
Enjoyment of food and encouragement (ENC)						RMSEA = 0.091 CFI = 0.964 SRMR = 0.020
Coupling parameter EF to ∆ENC	*γ*1	**0.07**	**0.02**	**0.11**	**.003**	
Coupling parameter ENC to ∆EF	*γ*2	**0.08**	**0.02**	**0.13**	**.006**	
Self‐feedback parameter EF	*β*1	−0.52	−0.47	−0.58	<.001	
Self‐feedback parameter ENC	*β*2	−0.59	−0.53	−0.64	<.001	
Covariance between EF and ENC at 15/16 months	*Ф*	0.04	0.02	0.06	.001	
Correlated change	*ρ*	0.02	0.00	0.04	.036	
Satiety responsiveness and encouragement (ENC)						RMSEA = 0.114 CFI = 0.942 SRMR = 0.025
Coupling parameter SR to ∆ENC	*γ*1	−0.01	−0.06	0.03	.590	
Coupling parameter ENC to ∆SR	*γ*2	0.01	−0.04	0.06	.574	
Self‐feedback parameter SR	*β*1	−0.59	−0.54	−0.64	<.001	
Self‐feedback parameter ENC	*β*2	−0.58	−0.53	−0.63	<.001	
Covariance between SR and ENC at 15/16 months	*Ф*	0.01	−0.02	0.03	.535	
Correlated change	*ρ*	0.01	−0.01	0.02	.312	
Slowness in eating and encouragement (ENC)						RMSEA = 0.121 CFI = 0.905 SRMR = 0.027
Coupling parameter SE to ∆ENC	*γ*1	0.01	−0.03	0.04	.668	
Coupling parameter ENC to ∆SE	*γ*2	0.05	−0.01	0.10	.092	
Self‐feedback parameter SE	*β*1	−0.67	−0.62	−0.73	<.001	
Self‐feedback parameter ENC	*β*2	−0.58	−0.54	−0.61	<.001	
Covariance between SE and ENC at 15/16 months	*Ф*	−0.002	−0.02	0.02	.844	
Correlated change	*ρ*	0.02	0.00	0.03	.049	
Food fussiness and encouragement (ENC)						CFI = 0.959 RMSEA = 0.096 SRMR = 0.021
Coupling parameter FF to ∆ENC	*γ*1	−0.04	−0.08	0.00	.077	
Coupling parameter ENC to ∆FF	*γ*2	−0.04	−0.11	0.03	.268	
Self‐feedback parameter FF	*β*1	−0.50	−0.44	−0.56	<.001	
Self‐feedback parameter ENC	*β*2	−0.58	−0.53	−0.63	<.001	
Covariance between FF and ENC at 15/16 months	*Ф*	−0.004	−0.03	0.02	.765	
Correlated change	*ρ*	0.00	−0.01	0.02	.648	

*Explanation of the parameters: Coupling parameters (γ1 and γ2)* – reflects the extent to which baseline levels in one domain (e.g. eating behaviour or parent feeding) predicts the rate or degree of change in the other domain (e.g. parent feeding or eating behaviour). A positive relationship for the *eating behaviour to parent feeding coupling parameter (γ1)* would indicate that higher eating behaviour trait scores at 15/16 months predicted greater increases in the feeding practice from 15/16 months to 5 years. A positive relationship for the *parent feeding to eating behaviour coupling parameter (γ2)* would indicate that higher parental feeding scores at 15/16 months predicted greater increases in the eating behaviour trait from 15/16 months to 5 years. *Self‐feedback parameters (β1 and β2)* – reflects the extent to which baseline levels in one domain (e.g. eating behaviour or parent feeding) influences change in the same domain (e.g. eating behaviour or parental feeding). The self‐feedback parameter is often negative which reflects regression towards the mean or ceiling effects and should not be overinterpreted. *Covariance at 15/16 months (Φ)* – reflects the covariance between feeding practices and eating behaviour at baseline (15/16 months). *Correlated change (ρ)* ‐ reflects the degree to which PFPs changes and eating behaviour changes co‐occur after taking into account the coupling parameters.

^a^ All models were adjusted for clustering within families and covariates; age of child at measurement (15/16 months and 5 years), SES, gestational age, sex of child. Significant results for the parameters of interest in this study are shown in bold.

^b^ Model fit indices were calculated, with cut‐offs in parentheses indicating acceptable to good fit: Comparative Fit Index (CFI ≥ 0.90), Root Mean Square Error of Approximation (RMSEA ≤ 0.10) and Standardised Root Mean Square Residuals (SRMR ≤ 0.08).

**Table 5:** Parameter estimates for bivariate latent change model^a^ between emotional feeding and five child eating behaviour traits

Parameter^a^		Estimate	95% CI		*p*‐Value	Fit statistics^b^
Emotional overeating (EOE) and emotional feeding (EMO)						CFI = 0.990 RMSEA = 0.049 SRMR = 0.013
Coupling parameter EOE to change in EMO	*γ*1	0.08	0.02	0.14	.011	
Coupling parameter EMO to ∆EOE	*γ*2	**0.12**	**0.07**	**0.16**	**<.001**	
Self‐feedback parameter EOE	*β*1	−0.81	−0.75	−0.87	<.001	
Self‐feedback parameter EMO	*β*2	−0.69	−0.64	−0.74	<.001	
Covariance between EOE and EMO at 15/16 months	*Ф*	0.14	0.11	0.17	<.001	
Correlated change	*ρ*	0.09	0.07	0.11	<.001	
Food responsiveness (FR) and emotional feeding (EMO)						CFI = 0.995 RMSEA = 0.034 SRMR = 0.009
Coupling parameter FR to ∆EMO	*γ*1	0.05	0.00	0.09	.044	
Coupling parameter EMO to ∆FR	*γ*2	**0.11**	**0.05**	**0.17**	**<.001**	
Self‐feedback parameter FR	*β*1	−0.60	−0.55	−0.66	<.001	
Self‐feedback parameter EMO	*β*2	−0.68	−0.63	−0.73	<.001	
Covariance between FR and EMO at 15/16 months	*Ф*	0.13	0.10	0.17	<.001	
Correlated change over 4 years	*ρ*	0.05	0.03	0.07	<.001	
Enjoyment of food (EF) and emotional feeding (EMO)						CFI = 0.979 RMSEA = 0.067 SRMR = 0.015
Coupling parameter EF to ∆EMO	*γ*1	0.00	−0.05	0.05	.980	
Coupling parameter EMO to ∆EF	*γ*2	0.01	−0.04	0.06	.747	
Self‐feedback parameter EF	*β*1	−0.52	−0.45	−0.57	<.001	
Self‐feedback parameter EMO	*β*2	−0.67	−0.62	−0.72	<.001	
Covariance between EF and EMO at 15/16 months	*Ф*	−0.02	−0.04	0.01	.249	
Correlated change	*ρ*	−0.00	−0.02	0.01	.791	
Satiety responsiveness and emotional feeding (EMO)						CFI = 0.972 RMSEA = 0.076 SRMR = 0.017
Coupling parameter SR to ∆EMO	*γ*1	0.01	−0.03	0.06	.548	
Coupling parameter EMO to ∆SR	*γ*2	0.03	−0.01	0.08	.151	
Self‐feedback parameter SR	*β*1	−0.59	−0.54	−0.65	<.001	
Self‐feedback parameter EMO	*β*2	−0.67	−0.62	−0.72	<.001	
Covariance between SR and EMO at 15/16 months	*Ф*	0.02	−0.01	0.04	.264	
Correlated change	*ρ*	0.02	0.01	0.04	.003	
Slowness in eating and emotional feeding (EMO)						CFI = 0.967 RMSEA = 0.071 SRMR = 0.017
Coupling parameter SE to ∆EMO	*γ*1	0.02	−0.02	0.06	.299	
Coupling parameter EMO to ∆SE	*γ*2	0.05	0.00	0.11	.040	
Self‐feedback parameter SE	*β*1	−0.67	−0.61	−0.73	<.001	
Self‐feedback parameter EMO	*β*2	−0.67	−0.62	−0.72	<.001	
Covariance between SE and EMO at 15/16 months	*Ф*	−0.02	−0.05	0.01	.162	
Correlated change	*ρ*	0.02	0.01	0.04	.008	
Food fussiness and emotional feeding (EMO)						CFI = 0.993 RMSEA = 0.037 SRMR = 0.009
Coupling parameter FF to ∆EMO	*γ*1	0.04	−0.00	0.09	.069	
Coupling parameter EMO to ∆FF	*γ*2	0.03	−0.03	0.09	.337	
Self‐feedback parameter FF	*β*1	−0.50	−0.44	−0.56	<.001	
Self‐feedback parameter EMO	*β*2	−0.67	−0.63	−0.72	<.001	
Covariance between FF and EMO at 15/16 months	*Ф*	0.04	0.01	0.07	.009	
Correlated change	*ρ*	0.02	0.00	0.04	.021	

^a^ All models were adjusted for clustering within families and covariates; age of child at measurement (15/16 months and 5 years), SES, gestational age, sex of child. Significant results for the parameters of interest in this study are shown in bold.

^b^ Model fit indices were calculated, with cut‐offs in parentheses indicating acceptable to good fit: Comparative Fit Index (CFI ≥ 0.90), Root Mean Square Error of Approximation (RMSEA ≤ 0.10) and Standardised Root Mean Square Residuals (SRMR ≤ 0.08).

### Reciprocal relationships between PFPs and child eating behaviours

#### Nonresponsive feeding practices

**Figure 2:**
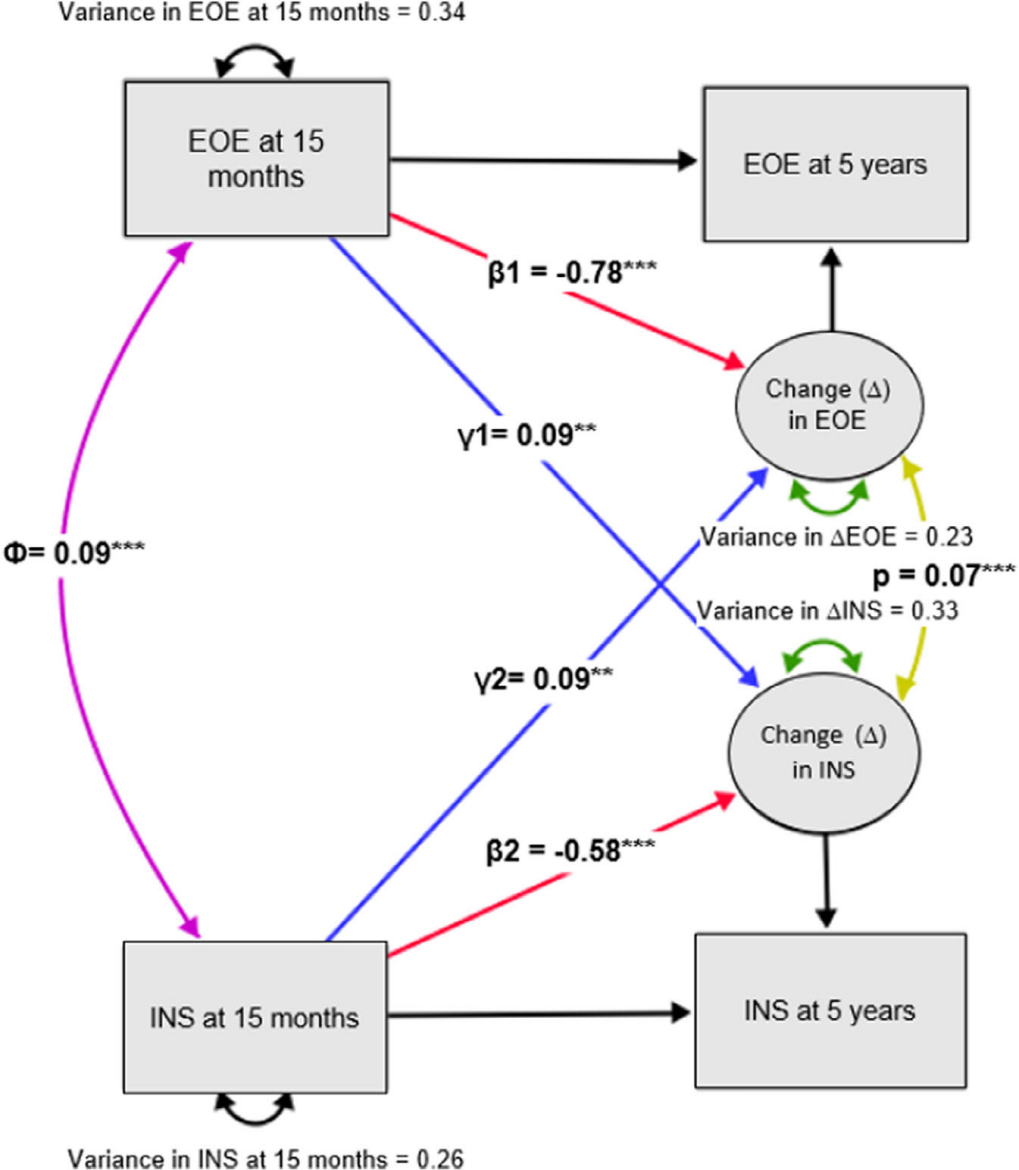
Bivariate latent change score model showing reciprocal relationships between instrumental feeding (INS) and emotional overeating (EOE). Circles indicate latent variables and squares indicate observed variables. Purple and yellow lines indicate cross‐domain undirected associations, blue lines indicate directed cross‐domain regressions, red lines indicate directed within domain associations, black lines indicate associations where the parameter estimates were fixed to 1. Analyses were adjusted for clustering within families and covariates; age of child at measurement (15/16 months and 5 years), SES, gestational age, sex of child. CFI: 0.99; RMSEA 0.05; SRMR: 0.01. **p < .01; ***p < .001 [Color figure can be viewed at wileyonlinelibrary.com]

Prospectively, a positive coupling parameter was observed between instrumental feeding and change in EOE; with higher instrumental feeding associated with greater increases in EOE from 15/16 months to 5 years (*β* = .09; 95% CI = 0.03, 0.15; *p* = .004; see Figure 2, Table 3). At the same time, higher EOE at 15/16 months elicited greater increases in instrumental feeding from 15 months to 5 years (*β* = .09; 95% CI = 0.03, 0.15; *p* = .007). Effect sizes were small.

#### Responsive feeding practices

**Figure 3:**
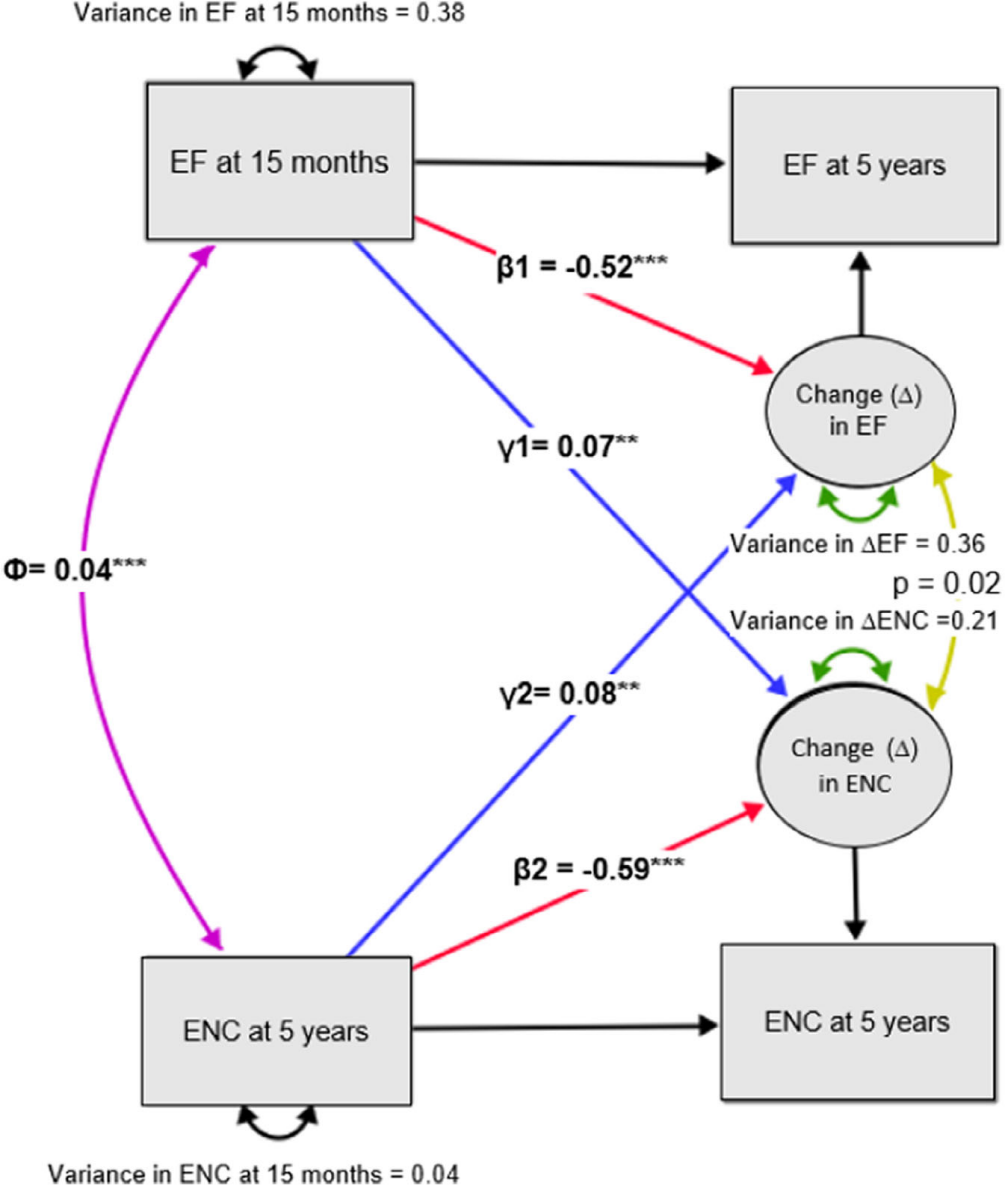
Bivariate latent change score model showing reciprocal relationships between encouragement to eat fruits and vegetables (ENC) and enjoyment of food (EF). Circles indicate latent variables and squares indicate observed variables. Purple and yellow lines indicate cross‐domain undirected associations, blue lines indicate directed cross‐domain regressions, red lines indicate directed within domain associations, black lines indicate associations where the parameter estimates were fixed to 1. Analyses were adjusted for clustering within families and covariates; age of child at measurement (15 months and 5 years), SES, gestational age, sex of child [Color figure can be viewed at wileyonlinelibrary.com]

Prospectively, higher encouragement to eat nutritious foods (e.g. fruits and vegetables) at 15/16 months predicted greater increases in enjoyment of food from 15/16 months to 5 years (*β* = .08; 95% CI = 0.02, 0.13; *p* = .006). At the same time, higher enjoyment of food at 15/16 months was associated with greater increases in encouragement to eat nutritious foods between 15/16 months and 5 years (*β* = .07; 95% CI = 0.02, 0.11; *p* = .003; Figure 3, Table 4). Effect sizes were small.

### Relationships from parent to child

Five relationships suggested PFPs influenced the development of child eating behaviours insofar as the prospective paths from parent to child were significant, while the prospective paths from child to parent were not. Effect sizes were small for all associations.

#### Nonresponsive feeding practices

Prospectively, greater emotional feeding at 15/16 months predicted greater increases in food responsiveness from 15/16 months to 5 years (Table 5; *β* = .11; 95% CI = 0.05, 0.17; *p* < .001). Prospectively, we observed a positive coupling parameter between emotional feeding and change in EOE; higher emotional feeding at 15/16 months was associated with greater increases in EOE from 15/16 months to 5 years (*β* = .12; 95% CI = 0.07, 0.16; *p* < .001; Table 5). Positive coupling parameters were observed between pressure to eat and change in EOE and also change in slowness in eating. Children who experienced more pressure to eat at 15/16 months showed greater increases in emotional overeating (Table S4; *β* = .07; 95% CI = 0.03, 0.11; *p* < .001) and greater increases in slowness in eating from 15/16 months to 5 years (Table S4; *β* = .12; 95% CI = 0.07, 0.17; *p* < .001).

#### Responsive feeding practices

Higher parental monitoring over food intake at 15/16 months was associated with greater increases in enjoyment of food from 15/16 months to 5 years (Table S7; *β* = .06; 95% CI = 0.02, 0.09; *p* = .002).

### Relationships from child to parent

Three relationships suggested child eating behaviours influenced the development of PFPs insofar as the prospective paths from child to parent were significant, while the prospective paths from parent to child were not. Effect sizes were small for all associations.

#### Nonresponsive feeding practices

Higher food responsiveness at 15/16 months was associated with greater increases in instrumental feeding from 15/16 months to 5 years (Table 3; *β* = .12; 95% CI = 0.07, 0.17; *p* < .001) and greater increases in pressure to eat from 15/16 months to 5 years (Table S3; *β* = .08, 95% CI = 0.03, 0.12; *p* = .001).

#### Responsive feeding practices

A positive coupling parameter was observed between enjoyment of food and change in modelling of healthy food intake (*β* = .10; 95% CI = 0.04, 0.16; *p* = .002; see Table S2), with higher enjoyment of food associated with greater increases in modelling from 15/16 months to 5 years.

The findings of the analyses using MLMV to deal with missing follow‐up data (*n* = 3,787) largely mirrored the main analysis, with comparable effect sizes observed (see Tables S8–S15). The main difference was that the relationship path from emotional overeating to emotional feeding reached significance (*β* = .08, 0.02–0.14, *p* = .008), although the effect size was identical to that observed in the main analyses. Model fit in the sensitivity analysis was improved slightly across SEM models.

## Discussion

This study is the first to investigate the directionality of associations between a broad range of PFPs and eating behaviours in preschool aged children. The findings reveal a reciprocal relationship between instrumental feeding and emotional overeating. These findings indicate that the nonresponsive, controlling feeding practice of instrumental feeding nurtures increases in emotional overeating, but at the same time, it was also used as a natural response to a child expressing a tendency to emotionally overeat. Additionally, we observed a reciprocal relationship between parental encouragement to eat healthy foods and children's enjoyment of food. Our findings indicate that the extent to which a parent encourages their child to eat healthy foods in toddlerhood influences their child's enjoyment of food over time. Equally, the amount a child enjoys food in toddlerhood influences the extent to which a parent encourages their child to eat nutritious food (e.g. fruits and vegetables). In addition to these reciprocal relationships, we also observed child‐ and parent‐driven relationships. In particular, pressuring a child to eat when they are not hungry predicted greater increases in emotional overeating and slowness in eating. Greater emotional feeding predicted greater increase in two key eating behaviours that characterise a greater interest in and enthusiasm for food (food responsiveness and emotional overeating). Greater monitoring of food intake predicted greater increases in enjoyment of food. Child‐driven relationships observed in this study were that higher food responsiveness predicted greater increases in the use of instrumental feeding and pressure to eat from toddlerhood to early childhood. Greater enjoyment of food also predicted greater parental modelling. However, all effect sizes were small, and as such the findings should not be overinterpreted. It is likely that the effects were small as child eating behaviours and feeding practices are influenced by a variety of factors (e.g. food availability, genetics).

Our findings revealed a reciprocal relationship observed between instrumental feeding and children's emotional overeating between 15/16 months and 5 years. These findings support previous longitudinal research which revealed that instrumental feeding (i.e. the use of food as a reward or contingency) was associated with higher emotional overeating in children over time (Farrow, Haycraft, & Blissett, 2015; Jansen et al., 2020; Steinsbekk et al., 2016). Our findings extend this by suggesting that effects are also child‐driven, with parents using instrumental feeding in response to their child expressing greater emotional overeating. Such child‐driven effects were observed in a large longitudinal population‐based study of children aged 4–9 years old (*n* = 3,642) which found that parents tended to use food as a reward in response to their child exhibiting eating behaviours that characterise a greater interest in and enthusiasm for food such as higher emotional overeating and higher food responsiveness (Jansen et al., 2020). Our findings mirror these child‐driven effects, with higher food responsiveness at 15/16 months predicting greater increases in instrumental feeding from 15/16 months to 5 years. These findings suggest that parents of children who are more food responsive appear to use food to control their child's behaviour (e.g. as a reward or contingency) more often, which may be because they perceive that their child is likely to respond positively to the sight and smell—or even the thought—of the reward food. Observational research has suggested that the types of foods that are typically offered in the context of instrumental feeding are palatable and energy‐dense foods (Raaijmakers, Gevers, Teuscher, Kremers, & van Assema, 2014), and as such, evidence suggests that use of food as a reward may increase a child's preference for the reward food and the child may also learn that eating could be a way to cope with negative emotional stressors.

Reciprocity was observed between parental encouragement to eat healthy foods (e.g. fruits and vegetables) and children's enjoyment of food from 15/16 months to 5 years. These findings extend previous research conducted in a Norwegian cohort (*n* = 797) which demonstrated that greater parental encouragement to eat healthy foods at age 6 predicted increases in enjoyment of food over a 2‐year period (Steinsbekk et al., 2016). Contrary to the findings in our study, the reverse relationship was not observed by Steinsbekk et al. These differences in findings may be due to differences in age between the two cohorts, the length of follow‐up, or sample size. It has been proposed that greater enjoyment of food may result from children enjoying the shared experience of eating with their parents (Gregory, Paxton, & Brozovic, 2010). Furthermore, a parent may be more inclined to encourage their child to eat fruits and vegetables and wide variety of foods if they expect their child to be receptive to this. This may also be true for modelling, as we also observed that greater enjoyment of food predicted increases in modelling from 15/16 months to 5 years. Previous research has associated greater enjoyment of food with the consumption of highly palatable energy‐dense foods (Webber, Cooke, Hill, & Wardle, 2010) and higher adiposity outcomes (Kininmonth et al., 2021). However, it is important to note that although enjoyment of food is typically deemed a ‘food approach’ behaviour, enjoying a wide variety of foods is an important part of a child developing a healthy relationship with food, and has also been associated with greater liking and consumption of fruits and vegetables (Cooke et al., 2004; Fildes et al., 2015). These findings suggest that encouragement to eat healthy foods such as fruits and vegetables, and a wide variety of foods, may be an important feeding practice that plays a role in shaping a child's enjoyment of food, but also that it is used in response to a child's existing enjoyment. As such, this reciprocity should be taken into consideration when developing interventions focussing on PFPs and children's eating behaviours.

Parent‐driven relationships were also observed, with our findings revealing that the use of food to soothe a child's emotions (emotional feeding) predicted greater increases in two eating behaviour traits that characterise a greater interest in and enthusiasm for food (food responsiveness and emotional overeating) between 15/16 months to 5 years. These findings extend previous cross‐sectional and longitudinal research which found that parents who use food to soothe (emotional feeding) may encourage their children to overeat in response to negative emotions (e.g. emotional overeating) and be more responsive to the sight, smell and thought of palatable foods (Rodgers et al., 2013; Steinsbekk et al., 2016). Importantly, this study suggests that this relationship is established very early in life, within the period during which toddlers are still making the transition onto family meals. These findings align with previous research which has shown that emotional feeding (feeding in response to emotional distress) may encourage a child to eat for reasons other than hunger (e.g. external stimuli, emotions; Blissett, Haycraft, & Farrow, 2010; Steinsbekk et al., 2016).

### Implications and future directions

Taken together, the findings indicate that food responsiveness and emotional overeating are two key eating behaviour traits that appear to be most amenable to modification by PFPs and that emotional feeding and instrumental feeding are key PFPs that could be targeted as part of a feeding intervention for parents of children during the preschool formative years. These findings offer promise as findings from twin studies have outlined the importance of the shared environment in shaping individual differences in emotional overeating (87% of variance explained by shared environmental effects at 15 months and 93% at 5 years) and food responsiveness (30% at 3 months) in early childhood (Herle, Fildes, Steinsbekk, Rijsdijk, & Llewellyn, 2017; Llewellyn, van Jaarsveld, Johnson, Carnell, & Wardle, 2010). To our knowledge, only a small number of interventions have focussed on modifying PFPs and examined the effects on children's eating behaviours. One Australian‐based intervention called NOURISH has provided promising results in this area and focused on the earliest period of life when complementary feeding begins, during the first 16 months (Daniels et al., 2015). The NOURISH intervention led to reductions in nonresponsive feeding practices such as pressure to eat and restriction (Daniels et al., 2015), and reductions in emotional overeating and food responsiveness and increases in satiety responsiveness in the intervention group up to 3.5 years post‐intervention (Daniels et al., 2014; Magarey et al., 2016). Future research could utilise the learnings from this study and interventions such as NOURISH to develop targeted interventions for parents of children with a greater interest in and enthusiasm for food to support the development of children's healthy eating behaviours.

### Strengths and limitations

Strengths of this study include the large sample size, prospective analyses, the use of multiple, well‐established, psychometric measures of PFPs and child eating behaviour. Furthermore, the analytic approach used offers a unique examination of the relationship between eating behaviour traits and PFPs and goes above and beyond usual longitudinal analyses (Kievit et al., 2018). However, there are some limitations that should be acknowledged. Although this study used psychometric measures to assess PFPs and eating behaviour traits, these measures are parent‐reported and subjective in nature, which may introduce measurement error. However, previous research has shown good correspondence with more objective measures of eating behaviour (Carnell & Wardle, 2007a). Nonetheless, it is important to note that the internal consistency as indicated by Cronbach's alpha was low (<.6) for instrumental feeding at 15/16‐months. Furthermore, as is common with longitudinal studies, the sample was relatively affluent, with a higher proportion of mid‐high SES families compared to low SES families and the majority identifying as White‐British; therefore, the findings may not be representative of families from more deprived or ethnically diverse backgrounds and may only be generalisable to White‐British families. PFPs have been shown to differ by socioeconomic status and ethnicity (Cardel et al., 2012; Gross, Mendelsohn, Fierman, Racine, & Messito, 2012). Therefore, future research needs to replicate these findings in a more socioeconomically and ethnically diverse sample. Additionally, the cohort used was over 10 years old which may be considered a weakness as it is not as contemporary as other samples. However, the children were born in 2007 so were born into and reared in the modern, highly obesogenic environment and we do not have empirical evidence to suggest that feeding practices have changed in this period. Furthermore, Gemini is the most comprehensive UK‐based longitudinal birth cohort with repeated measures of eating behaviours and PFPs from early life available to the authors, which would address the research questions. This investigation provides a basis for other studies to replicate the findings using more contemporary samples. Another limitation of the study is that no correction for multiple testing was undertaken and instead results are provided in full, with 95% CI, to allow readers to apply correction for multiple testing using the approach they feel most appropriate. As no correction for multiple testing was applied, there may be inflated risk of type‐I errors. Finally, it is important to acknowledge that although the LCSM yields many benefits compared to cross‐lagged models (Kievit et al., 2018), the use of LCSM could be improved with a greater number of timepoints, as this makes it easier to capture more fine‐grained dynamic processes within and across domains (Hounkpatin, Boyce, Dunn, & Wood, 2018; Kievit et al., 2018).

## Conclusion

This is the first study to examine the directionality of relationships between PFPs and children's eating behaviour traits in the preschool formative years and indicates that the nonresponsive feeding practice instrumental feeding appears to nurture increases in emotional overeating in early childhood but at the same time is used as a ‘natural’ response to a child expressing greater emotional overeating tendencies. Additionally, the responsive feeding practice encouragement to eat nutritious foods appears to influence a child's enjoyment of food in early childhood, and at the same time, a child's enjoyment of food influences parents' use of encouragement to eat nutritious food. These findings provide important insights into the PFPs and eating behaviour traits that could be targeted as part of a tailored intervention to support parents of children during the preschool formative years to develop healthy eating patterns.

## Supporting information


**Table S1.** Parental feeding practice measures included in Gemini at 15/16 months.
**Table S2.** Descriptive statistics for sample with complete data on child eating behaviours and parental feeding practices at 15/16‐months and 5‐years (complete case sample) and those with missing data due to non‐completion.
**Table S3.** Parameter estimates for bivariate latent change model between modelling and six child eating behaviour traits.
**Table S4.** Parameter estimates for bivariate latent change model between pressure to eat and six child eating behaviour traits.
**Table S5.** Parameter estimates for bivariate latent change model between parent control over what and/or when child eats and six child eating behaviour traits.
**Table S6.** Parameter estimates for bivariate latent change model between covert restriction and six child eating behaviour traits.
**Table S7.** Parameter estimates for bivariate latent change model between monitoring and six child eating behaviour traits.
**Table S8.** Parameter estimates for bivariate latent change model between emotional feeding and six child eating behaviour traits using maximum likelihood with missing values (MLMV) *n* = 3,787.
**Table S9.** Parameter estimates for bivariate latent change model between instrumental feeding and six child eating behaviour traits using maximum likelihood with missing values (MLMV) *n* = 3,787.
**Table S10.** Parameter estimates for bivariate latent change model between modelling and six child eating behaviour traits using maximum likelihood with missing values (MLMV) *n* = 3,787.
**Table S11.** Parameter estimates for bivariate latent change model between encouragement and six child eating behaviour traits using maximum likelihood with missing values (MLMV) *n* = 3,787.
**Table S12.** Parameter estimates for bivariate latent change model between pressure to eat and six child eating behaviour traits using maximum likelihood with missing values (MLMV) *n* = 3,787.
**Table S13.** Parameter estimates for bivariate latent change model between parent control and six child eating behaviour traits using maximum likelihood with missing values (MLMV) *n* = 3,787.
**Table S14.** Parameter estimates for bivariate latent change model between covert restriction and six child eating behaviour traits using maximum likelihood with missing values (MLMV) *n* = 3,787.
**Table S15.** Parameter estimates for bivariate latent change model between monitoring and six child eating behaviour traits using maximum likelihood with missing values (MLMV) *n* = 3,787.
