# Supplementary material for: Reciprocal associations between parental feeding practices and child eating behaviours from toddlerhood to early childhood: bivariate latent change analysis in the Gemini cohort
Source: J Child Psychol Psychiatry. 2023 May 15;64(10):1432–45. doi: 10.1111/jcpp.13819 (PMC10953001; doi:10.1111/jcpp.13819)
Supplement: Supplementary file 1 — Table S1. Parental feeding practice measures included in Gemini at 15/16 months. Table S2. Descriptive statistics for sample with complete data on child eating behaviours and parental feeding practices at 15/16‐months and 5‐years (complete case sample) and those with missing data due to non‐completion. Table S3. Parameter estimates for bivariate latent change model between modelling and six child eating behaviour traits. Table S4. Parameter estimates for bivariate latent change model between pressure to eat and six child eating behaviour traits. Table S5. Parameter estimates for bivariate latent change model between parent control over what and/or when child eats and six child eating behaviour traits. Table S6. Parameter estimates for bivariate latent change model between covert restriction and six child eating behaviour traits. Table S7. Parameter estimates for bivariate latent change model between monitoring and six child eating behaviour traits. Table S8. Parameter estimates for bivariate latent change model between emotional feeding and six child eating behaviour traits using maximum likelihood with missing values (MLMV) n = 3,787. Table S9. Parameter estimates for bivariate latent change model between instrumental feeding and six child eating behaviour traits using maximum likelihood with missing values (MLMV) n = 3,787. Table S10. Parameter estimates for bivariate latent change model between modelling and six child eating behaviour traits using maximum likelihood with missing values (MLMV) n = 3,787. Table S11. Parameter estimates for bivariate latent change model between encouragement and six child eating behaviour traits using maximum likelihood with missing values (MLMV) n = 3,787. Table S12. Parameter estimates for bivariate latent change model between pressure to eat and six child eating behaviour traits using maximum likelihood with missing values (MLMV) n = 3,787. Table S13. Parameter estimates for bivariate latent change model between parent control an [file JCPP-64-1432-s001.docx]

**Supporting Information**

**Table S1.** Parental feeding practice measures included in Gemini at 15/16 months. The table details the original items from questionnaires, the items that were included at 15/16 months and any deletions and/or modifications are shown in red font.

| **Measures** | **Original items** | **Items**^a^ **included at 15/16 months** | **Item modifications at 5 years** |
| --- | --- | --- | --- |
| **Encouragement to eat (5 items; PFSQ)** (Wardle et al., 2002) | 1. I encourage my child to eat a wide variety of foods 2. I praise my child if s/he eats a new food 3. I praise my child if s/he eats what I give him/her 4. I encourage my child to enjoy his/her food 5. I present food in an attractive way to my child  - I encourage my child to taste each of the foods I serve at mealtimes - I encourage my child to try foods that s/he hasn’t tasted before - I encourage my child to look forward to the meal | 1. I encourage my child to eat a wide variety of foods 2. I praise my child if s/he eats a new food 3. I praise my child if he/she eats fruit or vegetables 4. I encourage my child to eat fruit or vegetables 5. I present fruit and vegetables in an attractive way to my child | No modifications (items same as 15 months). |
| **Instrumental feeding (4 items; PFSQ)**(Wardle et al., 2002) | 1. If my child misbehaves I withhold his/her favourite food 2. I use puddings as a bribe to get my child to eat his/her main course 3. I reward my child with something to eat when s/he is well-behaved 4. I use foods my child likes as a way to get him/her to eat “healthy” foods  - In order to get my child to behave him/herself I promise him/her something to eat | 1. If my child misbehaves, I withhold his/her favourite food 2. I use puddings as a bribe to get my child to eat his/her main course 3. I reward my child with something to eat when s/he is well behaved 4. I use foods my child likes as a way to get him/her to eat ‘healthy’ foods. | No modifications (items same as 15 months). |
| **Emotional feeding (5 items; PFSQ)** (Wardle et al., 2002) | 1. I give my child something to eat to make him/her feel better when s/he is feeling upset. 2. I give my child something to eat to make him/her feel better when s/he has been hurt. 3. I give my child something to eat if s/he is feeling bored. 4. I give my child something to eat to make him/her feel better when s/he is feeling angry 5. I give my child something to eat to make him/her feel better when s/he is worried | 1. I give my child something to eat to make him/her feel better when s/he is feeling upset 2. I give my child something to eat to make him/her feel better when s/he has hurt him/herself 3. I give my child something to eat to occupy him/her, e.g. when in company, shopping, or travelling. 4. I give my child something to eat to make him/her feel better when s/he is feeling grumpy 5. I give my child something to eat to make him/her feel better when s/he is feeling irritable | 1. No change. 2. I give my child something to eat to make him/her feel better when s/he has been hurt 3. I give my child something to eat when s/he is feeling bored 4. I give my child something to eat to make him/her feel better when s/he is feeling angry 5. I give my child something to eat to make him/her feel better when s/he is worried |
| **Parent control (5 items; PFSQ)** (Wardle et al., 2002) | 1. I allow my child to choose which foods to have for meals 2. I decide how many snacks my child should have 3. I let my child decide when s/he would like to have her meal 4. I let my child eat between meals whenever s/he wants 5. I decide what my child eats between meals  - I decide when it is time for my child to have a snack - I decide the times when my child eats his/her meals - I insist my child eats meals at the table - I allow my child to decide when s/he has had enough snacks to eat - I allow my child to wander around at meals | 1. I allow my child to choose which foods to have for meals 2. I decide how many snacks my child should have 3. I let my child decide when s/he would like to have his/her meal 4. I let my child eat between meals whenever s/he wants 5. I decide what my child eats between meals | No modifications (items same as 15 months). |
| **Pressure to eat (5 items; CFQ)**(Birch et al., 2001) | 1. My child should always eat all of the food on her plate 2. I have to be especially careful to make sure my child eats enough 3. If my child says “I’m not hungry”, I try to get her to eat anyway  - If I did not guide or regulate my child’s eating, she would eat much less than she should. | 1. My child should always eat all of the food I give him/her 2. I have to be especially careful to make sure my child eats enough 3. If my child thinks he/she isn’t hungry, I try to get him/her to eat anyway 4. If I did not guide or regulate my child’s eating, s/he would eat much less than s/he should 5. I insist my child eat some fruit or vegetables, even if s/he doesn’t want | No modifications (items same as 15 months). |
| **Monitoring (3 items; CFQ)**(Birch et al., 2001) | 1. How much do you keep track of the sweets (candy, ice cream, cake, pies, pastries) that your child eats? 2. How much do you keep track of the snack food (potato chips, Doritos, cheese puffs) that your child eats? 3. How much do you keep track of the high-fat foods that your child eats? | 1. I keep track of the sugary foods that my child eats 2. I keep track of the high fat foods that my child eats 3. I keep track of the foods my child’s been eating when he/she is not with me (e.g. with a childminder or family member) | No modifications (items same as 15 months). |
| **Modelling (4 items; CFPQ)**(Musher-Eizenman & Holub, 2007) | 1. I model healthy eating for my child by eating healthy foods myself 2. I try to eat healthy foods in front of my child, even if they are not my favourite 3. I try to show enthusiasm about eating healthy foods 4. I show my child how much I enjoy eating healthy foods | 1. I model healthy eating for my child by eating healthy foods myself 2. I try to eat healthy foods in front of my child, even if they are not my favourite 3. I try to show enthusiasm about eating healthy foods 4. I show my child how much I enjoy eating healthy foods | No modifications (items same as 15 months). |
| **Covert restriction (4 items; Overt-Covert Control)**(Ogden et al., 2006) | How often do you   1. Avoid going to cafes or restaurants with your children which sell unhealthy foods 2. Not buy foods that you would like to because you don’t want your children to have them 3. Try not to eat unhealthy foods when your children are around 4. Avoid buying biscuits and cakes and bringing them into the house | 1. I avoid going to cafes or restaurants with my child which sell unhealthy foods 2. I avoid buying unhealthy foods and bringing them into the house 3. I try not to eat unhealthy when my child is around 4. I ask other people not to feed my child unhealthy foods | No modifications (items same as 15 months). |
| ^a^All items are rated using a 5 point Likert-Scale, 1=’never’, 2=’rarely’, 3=’sometimes’, 4=‘often’, 5=’always’ | | | |
|  | | | |

**Table S2.** Descriptive statistics for sample with complete data on child eating behaviours and parental feeding practices at 15/16-months and 5-years (complete case sample) and those with missing data due to non-completion.

|  | *Complete case sample (n=1858)* | | *Participants with missing data* | |
| --- | --- | --- | --- | --- |
| *Sample characteristics* | *Mean (SD) or N (%)* | | *Mean (SD) or N (%)* | |
| Child Sex |  | | n=3787 | |
| *Female* | 955 (51.4) | | 1912 (50.5) | |
| Gestational Age (weeks) | 36.26 (2.44) | | 36.33 (2.47)  n=3775 | |
| Maternal age at twin birth (years) | 33.92 (4.59)^1^ | | 33.33 (5.04)  n=3779 | |
| Child Age at 15 months (months) | 15.64 (0.95) | | 15.82 (1.14)  n=3787 | |
| Child Age at five years (years) | 5.15 (0.13) | | 5.16 (0.13)  n=2051 | |
| SES composite score^2^ | 4.64 (1.25) | | 4.47 (1.31)  n=3599 | |
| Ethnicity |  | | n=3787 | |
| *White-British* | 1668 (89.9) | | 3349 (88.4) | |
| *White Other* | 110 (5.9) | | 226 (6.0) | |
| *Black, Black British, Caribbean, or African* | 14 (0.8) | | 54 (1.4) | |
| *Asian or Asian British* | 34 (1.8) | | 90 (2.4) | |
| *Mixed or multiple ethnic groups* | 28 (1.5) | | 58 (1.5) | |
| *Other ethnic group* | 4 (0.2) | | 10 (0.3) | |
| *Child eating behaviours* | ***15 months*** | ***5 years*** | ***15 months*** | ***5 years*** |
| Food Responsiveness | 2.23 (0.75) | 2.36 (0.74) | 2.28 (0.76)  n=3787 | 2.37 (0.74)  n=1981 |
| Emotional Overeating | 1.63 (0.58) | 1.56 (0.51) | 1.64 (0.58)  n=3787 | 1.56 (0.51)  n=1957 |
| Enjoyment of Food | 4.16 (0.62) | 3.89 (0.67) | 4.19 (0.62)  n=3787 | 3.88 (0.67)  n=1973 |
| Satiety Responsiveness | 2.68 (0.63) | 2.86 (0.62) | 2.69 (0.62)  n=3787 | 2.87 (0.61)  n=1977 |
| Slowness in Eating | 2.47 (0.65) | 2.83 (0.77) | 2.49 (0.65)  n=3787 | 2.83 (0.77)  n=1981 |
| *Parental feeding practices* | ***15 months*** | ***5 years*** | ***15 months*** | ***5 years*** |
| Emotional feeding | 2.00 (0.72) | 1.70 (0.55) | 2.05 (0.72)  n=3787 | 1.70 (0.56)  n=1981 |
| Pressure to eat | 2.21 (0.71) | 2.75 (0.66) | 2.24 (0.73)  n=3787 | 2.75 (0.67)  n=1981 |
| Instrumental feeding | 1.69 (0.51) | 2.33 (0.62) | 1.74 (0.52)  n=3787 | 2.34 (0.63)  n=1981 |
| Covert restriction | 3.07 (0.90) | 2.99 (0.80) | 3.07 (0.92)  n=3787 | 2.99 (0.81)  n=1979 |
| Control | 4.49 (0.45) | 4.15 (0.44) | 4.45 (0.48)  n=3787 | 4.14 (0.45)  n=1981 |
| Monitoring | 3.86 (0.98) | 3.55 (0.91) | 3.87 (0.96)  n=3787 | 3.55 (0.91)  n=1981 |
| Encouragement | 4.03 (0.62) | 4.14 (0.52) | 4.07 (0.61)  n=3787 | 4.14 (0.52)  n=1981 |
| Modelling | 3.40 (0.83) | 3.73 (0.70) | 3.41 (0.84)  n=3787 | 3.73 (0.70)  n=1979 |
| ^1^Missing data for 1 family, n= 928. ^2^SES composite scores ranged from 1.30-6.96 for complete case records. For participants with missing data SES composite scores ranged from 1.26 to 7.04. Lower scores on the composite reflect lower SES. | | | | |

**Table S3.** Parameter estimates for bivariate latent change model^1^ between modelling and six child eating behaviour traits.

| **Parameter** | | **Estimate** | **95% CI** | | **p-value** | **Fit statistics^2^** |
| --- | --- | --- | --- | --- | --- | --- |
| **Emotional overeating and modelling (MOD)** | | | | | | CFI = 0.939  RMSEA = 0.116  SRMR = 0.023 |
| Coupling: EOE at baseline to ∆MOD | γ1 | -0.03 | -0.09 | 0.04 | 0.407 |  |
| Coupling: MOD at baseline to ∆EOE | γ2 | 0.01 | -0.03 | 0.04 | 0.790 |  |
| Self-feedback: EOE at baseline to ∆EOE | β1 | -0.77 | -0.71 | -0.82 | <0.001 |  |
| Self-feedback: MOD at baseline to ∆MOD | β2 | -0.54 | -0.49 | -0.59 | <0.001 |  |
| Covariance between EOE and MOD at 15 months | Ф | 0.03 | 0.00 | 0.07 | 0.041 |  |
| Correlated change | ρ | -0.01 | -0.03 | 0.01 | 0.383 |  |
| **Food responsiveness and modelling (MOD)** | | | | | | CFI = 0.962  RMSEA = 0.106  SRMR = 0.021 |
| Coupling parameter FR to ∆MOD | γ1 | 0.02 | -0.03 | 0.07 | 0.486 |  |
| Coupling parameter MOD to ∆FR | γ2 | -0.04 | -0.08 | 0.01 | 0.117 |  |
| Self-feedback parameter FR | β1 | -0.58 | -0.52 | -0.63 | <0.001 |  |
| Self-feedback parameter MOD | β2 | -0.54 | -0.49 | -0.60 | <0.001 |  |
| Covariance between FR and MOD at 15 months | Ф | 0.03 | -0.01 | 0.07 | 0.134 |  |
| Correlated change | ρ | 0.01 | -0.02 | 0.03 | 0.535 |  |
| **Enjoyment of food and modelling (MOD)** | | | | | | CFI = 0.961  RMSEA = 0.106  SRMR = 0.022 |
| Coupling parameter EF to ∆MOD | γ1 | **0.10** | **0.04** | **0.16** | **0.002** |  |
| Coupling parameter MOD to ∆EF | γ2 | 0.05 | 0.00 | 0.09 | 0.031 |  |
| Self-feedback parameter EF | β1 | -0.53 | -0.47 | -0.58 | <0.001 |  |
| Self-feedback parameter MOD | β2 | -0.55 | -0.50 | -0.61 | <0.001 |  |
| Covariance between EF and MOD at 15 months | Ф | 0.07 | 0.05 | 0.10 | <0.001 |  |
| Correlated change | ρ | 0.02 | 0.00 | 0.04 | 0.080 |  |
| **Satiety responsiveness and modelling (MOD)** | | | | | | CFI = 0.940  RMSEA = 0.128  SRMR = 0.026 |
| Coupling parameter SR to ∆MOD | γ1 | -0.05 | -0.11 | 0.01 | 0.097 |  |
| Coupling parameter MOD to ∆SR | γ2 | -0.01 | -0.05 | 0.03 | 0.668 |  |
| Self-feedback parameter SR | β1 | -0.59 | -0.54 | -0.64 | <0.001 |  |
| Self-feedback parameter MOD | β2 | -0.54 | -0.49 | -0.60 | <0.001 |  |
| Covariance between SR and MOD at 15 months | Ф | 0.01 | -0.02 | 0.05 | 0.409 |  |
| Correlated change | ρ | 0.004 | -0.02 | 0.02 | 0.690 |  |
| **Slowness in eating and modelling (MOD)** | | | | | | CFI = 0.932  RMSEA = 0.123  SRMR = 0.026 |
| Coupling parameter SE to ∆MOD | γ1 | -0.05 | -0.10 | 0.01 | 0.118 |  |
| Coupling parameter MOD to ∆SE | γ2 | 0.01 | -0.04 | 0.05 | 0.746 |  |
| Self-feedback parameter SE | β1 | -0.67 | -0.61 | -0.73 | <0.001 |  |
| Self-feedback parameter MOD | β2 | -0.54 | -0.49 | -0.59 | <0.001 |  |
| Covariance between SE and MOD at 15 months | Ф | 0.02 | -0.01 | 0.05 | 0.139 |  |
| Correlated change | ρ | 0.003 | -0.02 | 0.03 | 0.807 |  |
| **Food fussiness and modelling (MOD)** | | | | | | CFI = 0.957  RMSEA = 0.110  SRMR = 0.022 |
| Coupling parameter FF to ∆MOD | γ1 | -0.04 | -0.09 | 0.02 | 0.164 |  |
| Coupling parameter MOD to ∆FF | γ2 | -0.06 | -0.00 | -0.11 | 0.042 |  |
| Self-feedback parameter FF | β1 | -0.50 | -0.44 | -0.56 | <0.001 |  |
| Self-feedback parameter MOD | β2 | -0.54 | -0.49 | -0.60 | <0.001 |  |
| Covariance between FF and MOD at 15 months | Ф | -0.03 | -0.06 | 0.01 | 0.118 |  |
| Correlated change | ρ | -0.001 | -0.03 | 0.03 | 0.929 |  |
| ^1^ All models were adjusted for clustering within families and covariates; age of child at measurement (15 months and 5 years), SES, gestational age, sex of child. Significant results for the parameters of interest in this study are shown in bold. ^2^Model fit indices were calculated, with cut-offs in parentheses indicating acceptable to good fit: Comparative Fit Index (CFI ≥ 0.90), Root Mean Square Error of Approximation (RMSEA ≤ 0.10) and Standardized Root Mean Square Residuals (SRMR ≤0.08). **Explanation of the parameters:**   - **Coupling parameters** **(γ1 & γ2)** – reflects the extent to which baseline levels in one domain (e.g. eating behaviour or parent feeding) predicts the rate or degree of change in the other domain (e.g. parent feeding or eating behaviour). A positive relationship for the **eating behaviour to parent feeding coupling parameter (γ1)** would indicate that higher eating behaviour scores at 15 months predicted greater increases in the parental feeding practice from 15 months to 5 years. A positive relationship for the **parent feeding to eating behaviour coupling parameter** **(γ2)** would indicate that higher parental feeding scores at 15 months predicted greater increases in the eating behaviour trait from 15m to 5 years. - **Self-feedback parameters (β1 and β2)** – reflects the extent to which baseline levels in one domain (e.g. eating behaviour or parent feeding) influences change in the same domain (e.g. eating behaviour or parental feeding). The self-feedback parameter is often negative which reflects regression towards the mean or ceiling effects and should not be overinterpreted. - **Covariance at 15 months (Φ)** – reflects the covariance between feeding practices and eating behaviour at 15 months. - **Correlated change (ρ)** - reflects the degree to which PFPs changes and eating behaviour changes co-occur after taking into account the coupling parameters. | | | | | | |

**Table S4.** Parameter estimates for bivariate latent change model^1^ between pressure to eat and six child eating behaviour traits.

| **Parameter** | | **Estimate** | **95% CI** | | **p-value** | **Fit statistics^2^** |
| --- | --- | --- | --- | --- | --- | --- |
| **Emotional overeating and pressure to eat (PRE)** | | | | | | CFI = 0.978  RMSEA = 0.057  SRMR = 0.013 |
| Coupling parameter EOE to ∆PRE | γ1 | 0.05 | -0.01 | 0.11 | 0.120 |  |
| Coupling parameter PRE to ∆EOE | **γ2** | **0.07** | **0.03** | **0.11** | **0.001** |  |
| Self-feedback parameter EOE | β1 | -0.77 | -0.71 | -0.82 | <0.001 |  |
| Self-feedback parameter PRE | β2 | -0.60 | -0.55 | -0.65 | <0.001 |  |
| Covariance between EOE and PRE at 15 months | Ф | 0.004 | -0.02 | 0.03 | 0.752 |  |
| Correlated change | ρ | 0.01 | -0.01 | 0.02 | 0.341 |  |
| **Food responsiveness and pressure to eat (PRE)** | | | | | | CFI = 0.992  RMSEA = 0.043  SRMR = 0.010  TLI = 0.939 |
| Coupling parameter FR to ∆PRE | **γ1** | **0.08** | **0.03** | **0.12** | **0.001** |  |
| Coupling parameter PRE to ∆FR | γ2 | 0.05 | -0.01 | 0.10 | 0.101 |  |
| Self-feedback parameter FR | β1 | -0.58 | -0.52 | -0.64 | <0.001 |  |
| Self-feedback parameter PRE | β2 | -0.60 | -0.55 | -0.65 | <0.001 |  |
| Covariance between FR and PRE at 15 months | Ф | -0.002 | -0.04 | 0.03 | 0.913 |  |
| Correlated change | ρ | -0.04 | -0.06 | -0.01 | 0.002 |  |
| **Enjoyment of food and pressure to eat (PRE)** | | | | | | CFI = 0.969  RMSEA = 0.088  SRMR = 0.016 |
| Coupling parameter EF to ∆PRE | γ1 | 0.004 | -0.06 | 0.07 | 0.898 |  |
| Coupling parameter PRE to ∆EF | γ2 | -0.02 | -0.07 | 0.03 | 0.423 |  |
| Self-feedback parameter EF | β1 | -0.52 | -0.46 | -0.58 | <0.001 |  |
| Self-feedback parameter PRE | β2 | -0.60 | -0.54 | -0.65 | <0.001 |  |
| Covariance between EF and PRE at 15 months | Ф | -0.12 | -0.15 | -0.09 | <0.001 |  |
| Correlated change | ρ | -0.10 | -0.07 | -0.12 | <0.001 |  |
| **Satiety responsiveness and pressure to eat (PRE)** | | | | | | CFI = 0.976  RMSEA = 0.073  SRMR = 0.018 |
| Coupling parameter SR to ∆PRE | γ1 | -0.04 | -0.10 | 0.02 | 0.172 |  |
| Coupling parameter PRE to ∆SR | γ2 | 0.03 | -0.02 | 0.07 | 0.232 |  |
| Self-feedback parameter SR | β1 | -0.60 | -0.55 | -0.65 | <0.001 |  |
| Self-feedback parameter PRE | β2 | -0.59 | -0.54 | -0.64 | <0.001 |  |
| Covariance between SR and PRE at 15 months | Ф | 0.09 | 0.06 | 0.12 | <0.001 |  |
| Correlated change | ρ | 0.09 | 0.06 | 0.11 | <0.001 |  |
| **Slowness in eating and pressure to eat (PRE)** | | | | | | CFI = 0.969  RMSEA = 0.088  SRMR = 0.016 |
| Coupling parameter SE to ∆PRE | γ1 | -0.01 | -0.07 | 0.04 | 0.628 |  |
| Coupling parameter PRE to ∆SE | **γ2** | **0.12** | **0.06** | **0.17** | **<0.001** |  |
| Self-feedback parameter SE | β1 | -0.70 | -0.64 | -0.75 | <0.001 |  |
| Self-feedback parameter PRE | β2 | -0.60 | -0.54 | -0.65 | <0.001 |  |
| Covariance between SE and PRE at 15 months | Ф | 0.07 | 0.05 | 0.10 | <0.001 |  |
| Correlated change | ρ | 0.14 | 0.12 | 0.17 | <0.001 |  |
| **Food fussiness and pressure to eat (PRE)** | | | | | | CFI = 0.992  RMSEA = 0.042  SRMR = 0.011 |
| Coupling parameter FF to ∆PRE | γ1 | 0.02 | -0.03 | 0.08 | 0.421 |  |
| Coupling parameter PRE to ∆FF | **γ2** | 0.02 | -0.04 | 0.08 | 0.579 |  |
| Self-feedback parameter FF | β1 | -0.50 | -0.44 | -0.56 | <0.001 |  |
| Self-feedback parameter PRE | β2 | -0.60 | -0.55 | -0.66 | <0.001 |  |
| Covariance between FF and PRE at 15 months | Ф | 0.13 | 0.10 | 0.16 | <0.001 |  |
| Correlated change | ρ | 0.10 | 0.07 | 0.13 | <0.001 |  |
| ^1^ All models were adjusted for clustering within families and covariates; age of child at measurement (15 months and 5 years), SES, gestational age, sex of child. Significant results for the parameters of interest in this study are shown in bold.^2^Model fit indices were calculated, with cut-offs in parentheses indicating acceptable to good fit: Comparative Fit Index (CFI ≥ 0.90), Root Mean Square Error of Approximation (RMSEA ≤ 0.10) and Standardized Root Mean Square Residuals (SRMR ≤0.08). **Explanation of the parameters:**   - **Coupling parameters** **(γ1 & γ2)** – reflects the extent to which baseline levels in one domain (e.g. eating behaviour or parent feeding) predicts the rate or degree of change in the other domain (e.g. parent feeding or eating behaviour). A positive relationship for the **eating behaviour to parent feeding coupling parameter (γ1)** would indicate that higher eating behaviour scores at 15m predicted greater increases in the parental feeding practice from 15 m to 5 years. A positive relationship for the **parent feeding to eating behaviour coupling parameter** **(γ2)** would indicate that higher parental feeding scores at 15m predicted greater increases in the eating behaviour trait from 15m to 5 years. - **Self-feedback parameters (β1 and β2)** – reflects the extent to which baseline levels in one domain (e.g. eating behaviour or parent feeding) influences change in the same domain (e.g. eating behaviour or parental feeding). The self-feedback parameter is often negative reflecting regression towards the mean or ceiling effects and should not be overinterpreted. - **Covariance at 15 months (Φ)** – reflects the covariance between feeding practices and eating behaviour at 15 months. - **Correlated change (ρ)** - reflects the degree to which PFPs changes and eating behaviour changes co-occur after taking into account the coupling parameters. | | | | | | |

**Table S5.** Parameter estimates for bivariate latent change model between parent control over what and/or when child eats and six child eating behaviour traits.

| **Parameter** | | **Estimate** | **95% CI** | | **p-value** | **Fit statistics** |
| --- | --- | --- | --- | --- | --- | --- |
| **Emotional overeating and parent control (CON)** | | | | | | CFI = 0.910  SRMR = 0.026  RMSEA = 0.122 |
| Coupling parameter EOE to ∆CON | γ1 | 0.02 | -0.03 | 0.06 | 0.464 |  |
| Coupling parameter CON to ∆EOE | γ2 | -0.08 | -0.01 | -0.15 | 0.024 |  |
| Self-feedback parameter EOE | β1 | -0.77 | -0.71 | -0.84 | <0.001 |  |
| Self-feedback parameter CON | β2 | -0.55 | -0.49 | -0.61 | <0.001 |  |
| Covariance between EOE and CON at 15 months | Ф | -0.04 | -0.06 | -0.03 | <0.001 |  |
| Correlated change | ρ | -0.02 | -0.01 | -0.03 | 0.001 |  |
| **Food responsiveness and parent control (CON)** | | | | | | CFI = 0.953  SRMR = 0.022  RMSEA = 0.103 |
| Coupling parameter FR to ∆CON | γ1 | 0.01 | -0.02 | 0.04 | 0.557 |  |
| Coupling parameter CON to ∆FR | γ2 | -0.04 | -0.13 | 0.05 | 0.373 |  |
| Self-feedback parameter FR | β1 | -0.58 | -0.52 | -0.64 | <0.001 |  |
| Self-feedback parameter CON | β2 | -0.55 | -0.49 | -0.61 | <0.001 |  |
| Covariance between FR and CON at 15 months | Ф | -0.03 | -0.05 | -0.01 | 0.005 |  |
| Correlated change | ρ | -0.02 | -0.04 | -0.01 | 0.008 |  |
| **Enjoyment of food and parent control (CON)** | | | | | | CFI = 0.954  SRMR = 0.023  RMSEA = 0.100 |
| Coupling parameter EF to ∆CON | γ1 | 0.05 | 0.01 | 0.09 | 0.018 |  |
| Coupling parameter CON to ∆EF | γ2 | 0.05 | -0.03 | 0.13 | 0.221 |  |
| Self-feedback parameter EF | β1 | -0.52 | -0.46 | -0.58 | <0.001 |  |
| Self-feedback parameter CON | β2 | -0.56 | -0.50 | -0.62 | <0.001 |  |
| Covariance between EF and CON at 15 months | Ф | 0.04 | 0.02 | 0.06 | <0.001 |  |
| Correlated change | ρ | 0.03 | 0.01 | 0.04 | <0.001 |  |
| **Satiety responsiveness and parent control (CON)** | | | | | | CFI = 0.917  SRMR = 0.028  RMSEA = 0.133 |
| Coupling parameter SR to ∆CON | γ1 | -0.02 | -0.06 | 0.02 | 0.282 |  |
| Coupling parameter CON to ∆SR | γ2 | -0.02 | -0.09 | 0.05 | 0.587 |  |
| Self-feedback parameter SR | β1 | -0.59 | -0.54 | -0.64 | <0.001 |  |
| Self-feedback parameter CON | β2 | -0.55 | -0.50 | -0.61 | <0.001 |  |
| Covariance between SR and CON at 15 months | Ф | -0.04 | -0.06 | -0.02 | <0.001 |  |
| Correlated change | ρ | -0.02 | -0.04 | -0.01 | <0.001 |  |
| **Slowness in eating and parent control (CON)** | | | | | | CFI = 0.905  SRMR = 0.027  RMSEA = 0.121 |
| Coupling parameter SE to ∆CON | γ1 | -0.03 | -0.06 | 0.01 | 0.160 |  |
| Coupling parameter CON to ∆SE | γ2 | -0.004 | -0.09 | 0.08 | 0.920 |  |
| Self-feedback parameter SE | β1 | -0.67 | -0.61 | -0.73 | <0.001 |  |
| Self-feedback parameter CON | β2 | -0.55 | -0.50 | -0.61 | <0.001 |  |
| Covariance between SE and CON at 15 months | Ф | -0.03 | -0.05 | -0.01 | 0.002 |  |
| Correlated change | ρ | -0.01 | -0.02 | 0.01 | 0.389 |  |
| **Food fussiness and parent control (CON)** | | | | | | CFI = 0.948  RMSEA =0.106  SRMR = 0.023 |
| Coupling parameter FF to ∆CON | γ1 | -0.03 | -0.06 | 0.01 | 0.131 |  |
| Coupling parameter CON to ∆FF | γ2 | 0.02 | -0.07 | 0.11 | 0.649 |  |
| Self-feedback parameter FF | β1 | -0.49 | -0.44 | -0.55 | <0.001 |  |
| Self-feedback parameter CON | β2 | -0.56 | -0.50 | -0.61 | <0.001 |  |
| Covariance between FF and CON at 15 months | Ф | -0.04 | -0.02 | -0.06 | <0.001 |  |
| Correlated change | ρ | -0.03 | -0.02 | -0.05 | <0.001 |  |
| ^1^ All models were adjusted for clustering within families and covariates; age of child at measurement (15 months and 5 years), SES, gestational age, sex of child. Significant results for the parameters of interest in this study are shown in bold. ^2^Model fit indices were calculated, with cut-offs in parentheses indicating acceptable to good fit: Comparative Fit Index (CFI ≥ 0.90), Root Mean Square Error of Approximation (RMSEA ≤ 0.10) and Standardized Root Mean Square Residuals (SRMR ≤0.08). **Explanation of the parameters:**   - **Coupling parameters** **(γ1 & γ2)** – reflects the extent to which baseline levels in one domain (e.g. eating behaviour or parent feeding) predicts the rate or degree of change in the other domain (e.g. parent feeding or eating behaviour). A positive relationship for the **eating behaviour to parent feeding coupling parameter (γ1)** would indicate that higher eating behaviour scores at 15 months predicted greater increases in the parental feeding practice from 15 months to 5 years. A positive relationship for the **parent feeding to eating behaviour coupling parameter** **(γ2)** would indicate that higher parental feeding scores at 15 months predicted greater increases in the eating behaviour trait from 15 months to 5 years. - **Self-feedback parameters (β1 and β2)** – reflects the extent to which baseline levels in one domain (e.g. eating behaviour or parent feeding) influences change in the same domain (e.g. eating behaviour or parental feeding). The self-feedback parameter is often negative which reflects regression towards the mean or ceiling effects and should not be overinterpreted. - **Covariance at 15 months (Φ)** – reflects the covariance between feeding practices and eating behaviour at 15 months. - **Correlated change (ρ)** - reflects the degree to which PFPs changes and eating behaviour changes co-occur after taking into account the coupling parameters. | | | | | | |

**Table S6.** Parameter estimates for bivariate latent change model between covert restriction and six child eating behaviour traits.

| **Parameter** | | **Estimate** | **95% CI** | | **p-value** | **Fit statistics** |
| --- | --- | --- | --- | --- | --- | --- |
| **Emotional overeating and Covert restriction (COV)** | | | | | | CFI =0.993  SRMR = 0.011  RMSEA = 0.045 |
| Coupling parameter EOE to ∆COV | γ1 | 0.04 | -0.03 | 0.11 | 0.227 |  |
| Coupling parameter COV to ∆EOE | γ2 | -0.02 | -0.05 | 0.02 | 0.379 |  |
| Self-feedback parameter EOE | β1 | -0.76 | -0.71 | -0.82 | <0.001 |  |
| Self-feedback parameter COV | β2 | -0.47 | -0.42 | -0.52 | <0.001 |  |
| Covariance between EOE and COV at 15 months | Ф | 0.02 | -0.01 | 0.06 | 0.133 |  |
| Correlated change | ρ | 0.00 | -0.02 | 0.02 | 1.000 |  |
| **Food responsiveness and Covert restriction (COV)** | | | | | | CFI =0.999  SRMR = 0.005  RMSEA = 0.013 |
| Coupling parameter FR to ∆COV | γ1 | 0.003 | -0.05 | 0.06 | 0.915 |  |
| Coupling parameter COV to ∆FR | γ2 | -0.01 | -0.05 | 0.03 | 0.634 |  |
| Self-feedback parameter FR | β1 | -0.58 | -0.52 | -0.64 | <0.001 |  |
| Self-feedback parameter COV | β2 | -0.47 | -0.42 | -0.52 | <0.001 |  |
| Covariance between FR and COV at 15 months | Ф | -0.02 | -0.07 | 0.02 | 0.264 |  |
| Correlated change | ρ | -0.01 | -0.04 | 0.02 | 0.512 |  |
| **Enjoyment of food and Covert restriction (COV)** | | | | | | CFI =0.991  SRMR = 0.012  RMSEA = 0.054 |
| Coupling parameter EF to ∆COV | γ1 | 0.01 | -0.06 | 0.07 | 0.817 |  |
| Coupling parameter COV to ∆EF | γ2 | 0.04 | 0.00 | 0.08 | 0.028 |  |
| Self-feedback parameter EF | β1 | -0.52 | -0.46 | -0.58 | <0.001 |  |
| Self-feedback parameter COV | β2 | -0.47 | -0.42 | -0.52 | <0.001 |  |
| Covariance between EF and COV at 15 months | Ф | 0.04 | 0.01 | 0.08 | 0.014 |  |
| Correlated change | ρ | 0.02 | -0.01 | 0.04 | 0.140 |  |
| **Satiety responsiveness and Covert restriction (COV)** | | | | | | CFI =0.985  SRMR = 0.015  RMSEA = 0.069 |
| Coupling parameter SR to ∆COV | γ1 | -0.03 | -0.09 | 0.03 | 0.335 |  |
| Coupling parameter COV to ∆SR | γ2 | -0.01 | -0.05 | 0.02 | 0.455 |  |
| Self-feedback parameter SR | β1 | -0.59 | -0.54 | -0.64 | <0.001 |  |
| Self-feedback parameter COV | β2 | -0.47 | -0.42 | -0.52 | <0.001 |  |
| Covariance between SR and COV at 15 months | Ф | 0.02 | -0.01 | 0.06 | 0.217 |  |
| Correlated change | ρ | 0.002 | -0.02 | 0.02 | 0.816 |  |
| **Slowness in eating and Covert restriction (COV)** | | | | | | CFI =0.985  SRMR = 0.015  RMSEA = 0.064 |
| Coupling parameter SE to ∆COV | γ1 | -0.04 | -0.09 | 0.01 | 0.147 |  |
| Coupling parameter COV to ∆SE | γ2 | -0.05 | -0.09 | -0.01 | 0.025 |  |
| Self-feedback parameter SE | β1 | -0.67 | -0.61 | -0.73 | <0.001 |  |
| Self-feedback parameter COV | β2 | -0.47 | -0.42 | -0.52 | <0.001 |  |
| Covariance between SE and COV at 15 months | Ф | 0.02 | -0.01 | 0.05 | 0.250 |  |
| Correlated change | ρ | -0.01 | -0.04 | 0.01 | 0.276 |  |
| **Food fussiness and Covert Restriction (COV)** | | | | | | CFI = 0.999  RMSEA =0.015  SRMR = 0.006 |
| Coupling parameter FF to ∆COV | γ1 | -0.03 | -0.09 | 0.03 | 0.284 |  |
| Coupling parameter COV to ∆FF | γ2 | -0.02 | -0.06 | 0.03 | 0.496 |  |
| Self-feedback parameter FF | β1 | -0.50 | -0.43 | -0.56 | <0.001 |  |
| Self-feedback parameter COV | β2 | -0.47 | -0.42 | -0.52 | <0.001 |  |
| Covariance between FF and COV at 15 months | Ф | -0.02 | -0.05 | 0.02 | 0.292 |  |
| Correlated change | ρ | -0.01 | -0.03 | 0.02 | 0.601 |  |
| ^1^ All models were adjusted for clustering within families and covariates; age of child at measurement (15 months and 5 years), SES, gestational age, sex of child. Significant results for the parameters of interest in this study are shown in bold. ^2^Model fit indices were calculated, with cut-offs in parentheses indicating acceptable to good fit: Comparative Fit Index (CFI ≥ 0.90), Root Mean Square Error of Approximation (RMSEA ≤ 0.10) and Standardized Root Mean Square Residuals (SRMR ≤0.08). **Explanation of the parameters:**   - **Coupling parameters** **(γ1 & γ2)** – reflects the extent to which baseline levels in one domain (e.g. eating behaviour or parent feeding) predicts the rate or degree of change in the other domain (e.g. parent feeding or eating behaviour). A positive relationship for the **eating behaviour to parent feeding coupling parameter (γ1)** would indicate that higher eating behaviour scores at 15 months predicted greater increases in the parental feeding practice from 15 months to 5 years. A positive relationship for the **parent feeding to eating behaviour coupling parameter** **(γ2)** would indicate that higher parental feeding scores at 15 months predicted greater increases in the eating behaviour trait from 15 months to 5 years. - **Self-feedback parameters (β1 and β2)** – reflects the extent to which baseline levels in one domain (e.g. eating behaviour or parent feeding) influences change in the same domain (e.g. eating behaviour or parental feeding). The self-feedback parameter is often negative which reflects regression towards the mean or ceiling effects and should not be overinterpreted. - **Covariance at 15 months (Φ)** – reflects the covariance between feeding practices and eating behaviour at 15 months. - **Correlated change (ρ)** - reflects the degree to which PFPs changes and eating behaviour changes co-occur after taking into account the coupling parameters. | | | | | | |

**Table S7.** Parameter estimates for bivariate latent change model between monitoring and six child eating behaviour traits.

| **Parameter** | | **Estimate** | **95% CI** | | **p-value** | **Fit statistics** |
| --- | --- | --- | --- | --- | --- | --- |
| **Emotional overeating and monitoring (MON)** | | | | | | CFI = 0.990  SRMR = 0.011  RMSEA = 0.043 |
| Coupling parameter EOE to ∆MON | γ1 | 0.02 | -0.07 | 0.10 | 0.681 |  |
| Coupling parameter MON to ∆EOE | γ2 | 0.01 | -0.02 | 0.04 | 0.492 |  |
| Self-feedback parameter EOE | β1 | -0.77 | -0.71 | -0.82 | <0.001 |  |
| Self-feedback parameter MON | β2 | -0.53 | -0.47 | -0.59 | <0.001 |  |
| Covariance between EOE and MON at 15 months | Ф | -0.02 | -0.05 | 0.02 | 0.383 |  |
| Correlated change | ρ | -0.001 | -0.03 | 0.02 | 0.917 |  |
| **Food responsiveness and monitoring (MON)** | | | | | | CFI = 1.000  SRMR = 0.005  RMSEA = 0.008 |
| Coupling parameter FR to ∆MON | γ1 | 0.03 | -0.03 | 0.10 | 0.323 |  |
| Coupling parameter MON to ∆FR | γ2 | 0.03 | -0.01 | 0.07 | 0.140 |  |
| Self-feedback parameter FR | β1 | -0.58 | -0.52 | -0.63 | <0.001 |  |
| Self-feedback parameter MON | β2 | -0.53 | -0.47 | -0.59 | <0.001 |  |
| Covariance between FR and MON at 15 months | Ф | -0.01 | -0.06 | 0.03 | 0.609 |  |
| Correlated change | ρ | -0.003 | -0.03 | 0.03 | 0.875 |  |
| **Enjoyment of food and monitoring (MON)** | | | | | | CFI = 0.990  SRMR = 0.012  RMSEA = 0.051 |
| Coupling parameter EF to ∆MON | γ1 | 0.07 | -0.00 | 0.15 | 0.053 |  |
| Coupling parameter MON to ∆EF | γ2 | **0.06** | **0.02** | **0.09** | **0.002** |  |
| Self-feedback parameter EF | β1 | -0.53 | -0.47 | -0.59 | <0.001 |  |
| Self-feedback parameter MON | β2 | -0.54 | -0.48 | -0.59 | <0.001 |  |
| Covariance between EF and MON at 15 months | Ф | 0.08 | 0.04 | 0.12 | <0.001 |  |
| Correlated change | ρ | 0.04 | 0.02 | 0.07 | 0.001 |  |
| **Satiety responsiveness and monitoring (MON)** | | | | | | CFI = 0.979  SRMR = 0.016  RMSEA = 0.072 |
| Coupling parameter SR to ∆MON | γ1 | -0.05 | -0.13 | 0.03 | 0.197 |  |
| Coupling parameter MON to ∆SR | γ2 | -0.02 | -0.05 | 0.01 | 0.228 |  |
| Self-feedback parameter SR | β1 | -0.59 | -0.54 | -0.64 | <0.001 |  |
| Self-feedback parameter MON | β2 | -0.53 | -0.47 | -0.59 | <0.001 |  |
| Covariance between SR and MON at 15 months | Ф | -0.01 | -0.05 | 0.03 | 0.533 |  |
| Correlated change | ρ | -0.02 | -0.05 | 0.00 | 0.091 |  |
| **Slowness in eating and monitoring (MON)** | | | | | | RMSEA = 0.064  CFI = 0.990  SRMR = 0.015 |
| Coupling parameter SE to ∆MON | γ1 | -0.08 | -0.15 | -0.01 | 0.033 |  |
| Coupling parameter MON to ∆SE | γ2 | -0.004 | -0.04 | 0.03 | 0.824 |  |
| Self-feedback parameter SE | β1 | -0.67 | -0.61 | -0.73 | <0.001 |  |
| Self-feedback parameter MON | β2 | -0.53 | -0.59 | -0.47 | <0.001 |  |
| Covariance between SE and MON at 15 months | Ф | -0.02 | -0.06 | 0.02 | 0.271 |  |
| Correlated change | ρ | -0.02 | -0.05 | 0.01 | 0.277 |  |
| **Food fussiness and monitoring (MON)** | | | | | | CFI = 0.999  RMSEA = 0.019  SRMR = 0.006 |
| Coupling parameter FF to ∆MON | γ1 | -0.03 | -0.10 | 0.04 | 0.381 |  |
| Coupling parameter MON to ∆FF | γ2 | -0.02 | -0.07 | 0.02 | 0.344 |  |
| Self-feedback parameter FF | β1 | -0.50 | -0.44 | -0.56 | <0.001 |  |
| Self-feedback parameter MON | β2 | -0.53 | -0.47 | -0.59 | <0.001 |  |
| Covariance between FF and MON at 15 months | Ф | -0.07 | -0.03 | -0.11 | 0.001 |  |
| Correlated change | ρ | -0.01 | -0.05 | 0.02 | 0.519 |  |
| ^1^ All models were adjusted for clustering within families and covariates; age of child at measurement (15 months and 5 years), SES, gestational age, sex of child. Significant results for the parameters of interest in this study are shown in bold. ^2^Model fit indices were calculated, with cut-offs in parentheses indicating acceptable to good fit: Comparative Fit Index (CFI ≥ 0.90), Root Mean Square Error of Approximation (RMSEA ≤ 0.10) and Standardized Root Mean Square Residuals (SRMR ≤0.08). **Explanation of the parameters:**   - **Coupling parameters** **(γ1 & γ2)** – reflects the extent to which baseline levels in one domain (e.g. eating behaviour or parent feeding) predicts the rate or degree of change in the other domain (e.g. parent feeding or eating behaviour). A positive relationship for the **eating behaviour to parent feeding coupling parameter (γ1)** would indicate that higher eating behaviour scores at 15 months predicted greater increases in the parental feeding practice from 15 months to 5 years. A positive relationship for the **parent feeding to eating behaviour coupling parameter** **(γ2)** would indicate that higher parental feeding scores at 15 months predicted greater increases in the eating behaviour trait from 15 months to 5 years. - **Self-feedback parameters (β1 and β2)** – reflects the extent to which baseline levels in one domain (e.g. eating behaviour or parent feeding) influences change in the same domain (e.g. eating behaviour or parental feeding). The self-feedback parameter is often negative which reflects regression towards the mean or ceiling effects and should not be overinterpreted. - **Covariance at 15 months (Φ)** – reflects the covariance between parental feeding practices and eating behaviour at 15 months. - **Correlated change (ρ)** - reflects the degree to which PFPs changes and eating behaviour changes co-occur after taking into account the coupling parameters. | | | | | | |

**Table S8.** Parameter estimates for bivariate latent change model^1^ between emotional feeding and six child eating behaviour traits using maximum likelihood with missing values (MLMV) n=3787.

| **Parameter** | | **Estimate** | **95% CI** | | **p-value** | **Fit statistics^2^** |
| --- | --- | --- | --- | --- | --- | --- |
| **Emotional overeating (EOE) and emotional feeding (EMO)** | | | | | | CFI = 0.992  RMSEA = 0.034 |
| Coupling parameter EOE to change in EMO | γ1 | **0.08** | **0.02** | **0.14** | **0.008** |  |
| Coupling parameter EMO to ∆EOE | γ2 | **0.12** | **0.07** | **0.16** | **<0.001** |  |
| Self-feedback parameter EOE | β1 | -0.80 | -0.75 | -0.86 | <0.001 |  |
| Self-feedback parameter EMO | β2 | -0.67 | -0.62 | -0.72 | <0.001 |  |
| Covariance between EOE and EMO at 15 months | Ф | 0.15 | 0.13 | 0.17 | <0.001 |  |
| Correlated change | ρ | 0.09 | 0.08 | 0.11 | <0.001 |  |
| **Food responsiveness (FR) and emotional feeding (EMO)** | | | | | | CFI = 0.995  RMSEA = 0.034 |
| Coupling parameter FR to ∆EMO | γ1 | 0.05 | 0.00 | 0.09 | 0.034 |  |
| Coupling parameter EMO to ∆FR | γ2 | **0.11** | **0.06** | **0.17** | **<0.001** |  |
| Self-feedback parameter FR | β1 | -0.61 | -0.56 | -0.67 | <0.001 |  |
| Self-feedback parameter EMO | β2 | -0.66 | -0.61 | -0.71 | <0.001 |  |
| Covariance between FR and EMO at 15 months | Ф | 0.13 | 0.10 | 0.15 | <0.001 |  |
| Correlated change over 4 years | ρ | 0.05 | 0.03 | 0.07 | <0.001 |  |
| **Enjoyment of food (EF) and emotional feeding (EMO)** | | | | | | CFI = 0.980  RMSEA = 0.048 |
| Coupling parameter EF to ∆EMO | γ1 | -0.004 | -0.05 | 0.04 | 0.878 |  |
| Coupling parameter EMO to ∆EF | γ2 | 0.01 | -0.04 | 0.06 | 0.709 |  |
| Self-feedback parameter EF | β1 | -0.52 | -0.47 | -0.57 | <0.001 |  |
| Self-feedback parameter EMO | β2 | -0.65 | -0.60 | -0.70 | <0.001 |  |
| Covariance between EF and EMO at 15 months | Ф | -0.02 | -0.04 | -0.002 | 0.027 |  |
| Correlated change | ρ | -0.004 | -0.02 | 0.01 | 0.634 |  |
| **Satiety responsiveness and emotional feeding (EMO)** | | | | | | CFI = 0.976  RMSEA = 0.052 |
| Coupling parameter SR to ∆EMO | γ1 | 0.02 | -0.02 | 0.07 | 0.261 |  |
| Coupling parameter EMO to ∆SR | γ2 | 0.03 | -0.02 | 0.07 | 0.240 |  |
| Self-feedback parameter SR | β1 | -0.59 | -0.54 | -0.64 | <0.001 |  |
| Self-feedback parameter EMO | β2 | -0.65 | -0.60 | -0.70 | <0.001 |  |
| Covariance between SR and EMO at 15 months | Ф | 0.02 | -0.01 | 0.04 | 0.008 |  |
| Correlated change | ρ | 0.03 | 0.01 | 0.04 | 0.008 |  |
| **Slowness in eating and emotional feeding (EMO)** | | | | | | CFI = 0.975  RMSEA = 0.046 |
| Coupling parameter SE to ∆EMO | γ1 | 0.03 | -0.02 | 0.07 | 0.233 |  |
| Coupling parameter EMO to ∆SE | γ2 | 0.05 | 0.00 | 0.10 | 0.062 |  |
| Self-feedback parameter SE | β1 | -0.67 | -0.61 | -0.73 | <0.001 |  |
| Self-feedback parameter EMO | β2 | -0.65 | -0.60 | -0.69 | <0.001 |  |
| Covariance between SE and EMO at 15 months | Ф | -0.003 | -0.02 | 0.02 | 0.752 |  |
| Correlated change | ρ | 0.02 | 0.01 | 0.04 | 0.009 |  |
| **Food fussiness and emotional feeding (EMO)** | | | | | | CFI = 0.995  RMSEA = 0.023 |
| Coupling parameter FF to ∆EMO | γ1 | 0.03 | -0.01 | 0.08 | 0.116 |  |
| Coupling parameter EMO to ∆FF | γ2 | 0.03 | -0.03 | 0.08 | 0.373 |  |
| Self-feedback parameter FF | β1 | -0.51 | -0.45 | -0.56 | <0.001 |  |
| Self-feedback parameter EMO | β2 | -0.65 | -0.60 | -0.70 | <0.001 |  |
| Covariance between FF and EMO at 15 months | Ф | 0.05 | 0.03 | 0.07 | <0.001 |  |
| Correlated change | ρ | 0.02 | 0.01 | 0.04 | 0.014 |  |
| ^1^Models adjusted for clustering within families and covariates; child age at measurements, SES, gestational age, child sex. Significant results for the parameters of interest in this study are shown in bold. ^2^Model fit indices were calculated, with cut-offs in parentheses indicating acceptable to good fit: Comparative Fit Index (CFI ≥ 0.90), Root Mean Square Error of Approximation (RMSEA ≤ 0.10). **Explanation of the parameters:**   - **Coupling parameters** **(γ1 & γ2)** – reflects the extent to which baseline levels in one domain (e.g. eating behaviour or parent feeding) predicts the rate or degree of change (∆) in the other domain (e.g. parent feeding or eating behaviour). A positive relationship for the **eating behaviour to parent feeding coupling parameter (γ1)** would indicate that higher eating behaviour scores at 15 months predicted greater increases in the parental feeding practice from 15 months to 5 years. A positive relationship for the **parent feeding to eating behaviour coupling parameter** **(γ2)** would indicate that higher parental feeding scores at 15 months predicted greater increases in the eating behaviour trait from 15m to 5 years. - **Self-feedback parameters (β1 and β2)** – reflects the extent to which baseline levels in one domain (e.g. eating behaviour or parent feeding) influences change in the same domain (e.g. eating behaviour or parental feeding). - **Covariance at 15 months (Φ)** – reflects the covariance between parental feeding practices and eating behaviour at 15m. - **Correlated change (ρ)** - reflects the degree to which PFPs changes and eating behaviour changes co-occur after taking into account the coupling parameters. A positive relationship for correlated change indicates that over the 4-year period, greater increases in the eating behaviour trait were also associated with greater increases in the parental feeding practice. | | | | | | |

**Table S9.** Parameter estimates for bivariate latent change model^1^ between instrumental feeding and six child eating behaviour traits using maximum likelihood with missing values (MLMV) n=3787.

| **Parameter** | | **Estimate** | **95% CI** | | **p-value** | **Fit statistics^2^** |
| --- | --- | --- | --- | --- | --- | --- |
| **Emotional overeating and instrumental feeding (INS)** | | | | | | CFI = 0.986  RMSEA = 0.034 |
| Coupling parameter EOE to ∆INS | γ1 | **0.09** | **0.03** | **0.15** | **0.004** |  |
| Coupling parameter INS to ∆EOE | γ2 | **0.09** | **0.03** | **0.15** | **0.005** |  |
| Self-feedback parameter EOE | β1 | -0.77 | -0.71 | -0.83 | <0.001 |  |
| Self-feedback parameter INS | β2 | -0.56 | -0.49 | -0.64 | <0.001 |  |
| Covariance EOE & INS at 15 months | Ф | 0.05 | 0.04 | 0.06 | <0.001 |  |
| Correlated change | ρ | 0.07 | 0.05 | 0.09 | <0.001 |  |
| **Food responsiveness and instrumental feeding (INS)** | | | | | | CFI = 0.994  RMSEA = 0.025 |
| Coupling parameter FR to ∆INS | γ1 | **0.13** | **0.08** | **0.17** | **<0.001** |  |
| Coupling parameter INS to ∆FR | γ2 | 0.09 | 0.02 | 0.17 | 0.018 |  |
| Self-feedback parameter FR | β1 | -0.59 | -0.54 | -0.65 | <0.001 |  |
| Self-feedback parameter INS | β2 | -0.57 | -0.50 | -0.65 | <0.001 |  |
| Covariance between FR and INS at 15 months | Ф | 0.06 | 0.04 | 0.07 | <0.001 |  |
| Correlated change | ρ | 0.08 | 0.05 | 0.10 | <0.001 |  |
| **Enjoyment of food and instrumental feeding (INS)** | | | | | | CFI = 0.971  RMSEA = 0.050 |
| Coupling parameter EF to ∆INS | γ1 | 0.01 | -0.04 | 0.07 | 0.646 |  |
| Coupling parameter INS to ∆EF | γ2 | 0.001 | -0.06 | 0.06 | 0.978 |  |
| Self-feedback parameter EF | β1 | -0.53 | -0.47 | -0.58 | <0.001 |  |
| Self-feedback parameter INS | β2 | -0.54 | -0.47 | -0.62 | <0.001 |  |
| Covariance between EF and INS at 15 months | Ф | -0.02 | -0.01 | -0.03 | 0.004 |  |
| Correlated change | ρ | -0.02 | -0.04 | 0.003 | 0.090 |  |
| **Satiety responsiveness and instrumental feeding (INS)** | | | | | | CFI = 0.969  RMSEA = 0.054 |
| Coupling parameter SR to ∆INS | γ1 | -0.04 | -0.10 | 0.02 | 0.183 |  |
| Coupling parameter INS to ∆SR | γ2 | -0.001 | -0.06 | 0.06 | 0.970 |  |
| Self-feedback parameter SR | β1 | -0.59 | -0.54 | -0.64 | <0.001 |  |
| Self-feedback parameter INS | β2 | -0.54 | -0.46 | -0.61 | <0.001 |  |
| Covariance between SR and INS at 15 months | Ф | 0.03 | 0.02 | 0.04 | <0.001 |  |
| Correlated change | ρ | 0.04 | 0.03 | 0.06 | <0.001 |  |
| **Slowness in eating and instrumental feeding (INS)** | | | | | | CFI = 0.968  RMSEA = 0.045 |
| Coupling parameter SE to ∆INS | γ1 | -0.02 | -0.75 | 0.03 | 0.448 |  |
| Coupling parameter INS to ∆SE | γ2 | 0.07 | 0.00 | 0.15 | 0.055 |  |
| Self-feedback parameter SE | β1 | -0.68 | -0.62 | -0.73 | <0.001 |  |
| Self-feedback parameter INS | β2 | -0.54 | -0.47 | -0.62 | <0.001 |  |
| Covariance between SE and INS at 15 months | Ф | 0.02 | 0.00 | 0.03 | 0.028 |  |
| Correlated change | ρ | 0.05 | 0.03 | 0.07 | <0.001 |  |
| **Food fussiness and instrumental feeding** | | | | | | CFI = 0.993  RMSEA = 0.026 |
| Coupling parameter FF to ∆INS | γ1 | 0.03 | -0.02 | 0.08 | 0.298 |  |
| Coupling parameter INS to ∆FF | γ2 | 0.04 | -0.04 | 0.13 | 0.307 |  |
| Self-feedback parameter FF | β1 | -0.51 | -0.45 | -0.57 | <0.001 |  |
| Self-feedback parameter INS | β2 | -0.55 | -0.48 | -0.62 | <0.001 |  |
| Covariance between FF and INS at 15 months | Ф | 0.06 | 0.04 | 0.07 | <0.001 |  |
| Correlated change | ρ | 0.07 | 0.04 | 0.09 | <0.001 |  |
| ^1^Models adjusted for clustering within families and covariates; child age at measurements, sex, SES, gestational age. Significant results for the parameters of interest in this study are shown in bold. ^2^Model fit indices were calculated, with cut-offs in parentheses indicating acceptable to good fit: Comparative Fit Index (CFI ≥ 0.90), Root Mean Square Error of Approximation (RMSEA ≤ 0.10). **Explanation of the parameters:**   - **Coupling parameters** **(γ1 & γ2)** – reflects the extent to which baseline levels in one domain (e.g. eating behaviour or parent feeding) predicts the rate or degree of change (∆) in the other domain (e.g. parent feeding or eating behaviour). A positive relationship for the **eating behaviour to parent feeding coupling parameter (γ1)** would indicate that higher eating behaviour scores at 15 months predicted greater increases in the parental feeding practice from 15 months to 5 years. A positive relationship for the **parent feeding to eating behaviour coupling parameter** **(γ2)** would indicate that higher parental feeding scores at 15 months predicted greater increases in the eating behaviour trait from 15 months to 5 years. - **Self-feedback parameters (β1 and β2)** – reflects the extent to which baseline levels in one domain (e.g. eating behaviour or parent feeding) influences change in the same domain (e.g. eating behaviour or parental feeding). The self-feedback parameter is often negative which reflects regression towards the mean or ceiling effects and should not be overinterpreted. - **Covariance at 15 months (Φ)** – reflects the covariance between parental feeding practices and eating behaviour at 15 months. - **Correlated change (ρ)** - reflects the degree to which PFPs changes and eating behaviour changes co-occur after taking into account the coupling parameters. A positive relationship for correlated change indicates that over the 4-year period, greater increases in the eating behaviour trait were also associated with greater increases in the parental feeding practice. | | | | | | |

**Table S10.** Parameter estimates for bivariate latent change model^1^ between modelling and six child eating behaviour traits using maximum likelihood with missing values (MLMV) n=3787.

| **Parameter** | | **Estimate** | **95% CI** | | **p-value** | **Fit statistics^2^** |
| --- | --- | --- | --- | --- | --- | --- |
| **Emotional overeating and modelling (MOD)** | | | | | | CFI = 0.945  RMSEA = 0.082 |
| Coupling: EOE at baseline to ∆MOD | γ1 | -0.03 | -0.10 | 0.03 | 0.273 |  |
| Coupling: MOD at baseline to ∆EOE | γ2 | 0.01 | -0.02 | 0.04 | 0.575 |  |
| Self-feedback: EOE at baseline to ∆EOE | β1 | -0.76 | -0.70 | -0.81 | <0.001 |  |
| Self-feedback: MOD at baseline to ∆MOD | β2 | -0.53 | -0.48 | -0.59 | <0.001 |  |
| Covariance between EOE and MOD at 15 months | Ф | 0.03 | 0.01 | 0.06 | 0.002 |  |
| Correlated change | ρ | -0.01 | -0.03 | 0.01 | 0.297 |  |
| **Food responsiveness and modelling (MOD)** | | | | | | CFI = 0.961  RMSEA = 0.077 |
| Coupling parameter FR to ∆MOD | γ1 | 0.01 | -0.03 | 0.06 | 0.602 |  |
| Coupling parameter MOD to ∆FR | γ2 | -0.04 | -0.08 | 0.01 | 0.111 |  |
| Self-feedback parameter FR | β1 | -0.59 | -0.53 | -0.64 | <0.001 |  |
| Self-feedback parameter MOD | β2 | -0.54 | -0.48 | -0.59 | <0.001 |  |
| Covariance between FR and MOD at 15 months | Ф | 0.02 | -0.01 | 0.04 | 0.317 |  |
| Correlated change | ρ | 0.004 | -0.02 | 0.03 | 0.754 |  |
| **Enjoyment of food and modelling (MOD)** | | | | | | CFI = 0.962  RMSEA = 0.75 |
| Coupling parameter EF to ∆MOD | γ1 | **0.09** | **0.03** | **0.15** | **0.005** |  |
| Coupling parameter MOD to ∆EF | γ2 | 0.05 | 0.01 | 0.09 | 0.017 |  |
| Self-feedback parameter EF | β1 | -0.53 | -0.48 | -0.59 | <0.001 |  |
| Self-feedback parameter MOD | β2 | -0.55 | -0.49 | -0.60 | <0.001 |  |
| Covariance between EF and MOD at 15 months | Ф | 0.05 | 0.03 | 0.07 | <0.001 |  |
| Correlated change | ρ | 0.02 | 0.00 | 0.04 | 0.053 |  |
| **Satiety responsiveness and modelling (MOD)** | | | | | | CFI = 0.946  RMSEA = 0.089 |
| Coupling parameter SR to ∆MOD | γ1 | -0.03 | -0.09 | 0.02 | 0.255 |  |
| Coupling parameter MOD to ∆SR | γ2 | -0.01 | -0.04 | 0.03 | 0.697 |  |
| Self-feedback parameter SR | β1 | -0.59 | -0.54 | -0.64 | <0.001 |  |
| Self-feedback parameter MOD | β2 | -0.54 | -0.49 | -0.59 | <0.001 |  |
| Covariance between SR and MOD at 15 months | Ф | 0.01 | -0.01 | 0.03 | 0.425 |  |
| Correlated change | ρ | 0.003 | -0.02 | 0.02 | 0.773 |  |
| **Slowness in eating and modelling (MOD)** | | | | | | CFI = 0.932  RMSEA = 0.123 |
| Coupling parameter SE to ∆MOD | γ1 | -0.02 | -0.08 | 0.03 | 0.409 |  |
| Coupling parameter MOD to ∆SE | γ2 | 0.00 | -0.04 | 0.04 | 0.954 |  |
| Self-feedback parameter SE | β1 | -0.67 | -0.62 | -0.73 | <0.001 |  |
| Self-feedback parameter MOD | β2 | -0.54 | -0.48 | -0.59 | <0.001 |  |
| Covariance between SE and MOD at 15 months | Ф | 0.03 | 0.004 | 0.05 | 0.023 |  |
| Correlated change | ρ | 0.00 | -0.02 | 0.02 | 0.986 |  |
| Food fussiness and modelling | | | | | | |
| Coupling parameter FF to ∆MOD | γ1 | -0.03 | -0.08 | 0.02 | 0.305 | CFI = 0.960  RMSEA = 0.076 |
| Coupling parameter MOD to ∆FF | γ2 | -0.07 | -0.02 | -0.12 | 0.011 |  |
| Self-feedback parameter FF | β1 | -0.51 | -0.45 | -0.57 | <0.001 |  |
| Self-feedback parameter MOD | β2 | -0.54 | -0.48 | -0.59 | <0.001 |  |
| Covariance between FF and MOD at 15 months | Ф | -0.04 | -0.06 | -0.01 | 0.007 |  |
| Correlated change | ρ | -0.002 | -0.03 | 0.03 | 0.905 |  |
| ^1^ Models adjusted for clustering within families and covariates; child age at measurements, sex, SES, gestational age. Significant results for the parameters of interest in this study are shown in bold. ^2^Model fit indices were calculated, with cut-offs in parentheses indicating acceptable to good fit: Comparative Fit Index (CFI ≥ 0.90), Root Mean Square Error of Approximation (RMSEA ≤ 0.10). **Explanation of the parameters:**   - **Coupling parameters** **(γ1 & γ2)** – reflects the extent to which baseline levels in one domain (e.g. eating behaviour or parent feeding) predicts the rate or degree of change in the other domain (e.g. parent feeding or eating behaviour). A positive relationship for the **eating behaviour to parent feeding coupling parameter (γ1)** would indicate that higher eating behaviour scores at 15 months predicted greater increases in the parental feeding practice from 15 months to 5 years. A positive relationship for the **parent feeding to eating behaviour coupling parameter** **(γ2)** would indicate that higher parental feeding scores at 15m predicted greater increases in the eating behaviour trait from 15m to 5 years. - **Self-feedback parameters (β1 and β2)** – reflects the extent to which baseline levels in one domain (e.g. eating behaviour or parent feeding) influences change in the same domain (e.g. eating behaviour or parental feeding). The self-feedback parameter is often negative which reflects regression towards the mean or ceiling effects and should not be overinterpreted. - **Covariance at 15 months (Φ)** – reflects the covariance between parental feeding practices and eating behaviour at 15 months. - **Correlated change (ρ)** - reflects the degree to which PFPs changes and eating behaviour changes co-occur after taking into account the coupling parameters. A positive relationship for correlated change indicates that over the 4-year period, greater increases in the eating behaviour trait were also associated with greater increases in the parental feeding practice. | | | | | | |

**Table S11.** Parameter estimates for bivariate latent change model^1^ between encouragement and six child eating behaviour traits using maximum likelihood with missing values (MLMV) n=3787.

| **Parameter** | | **Estimate** | **95% CI** | | **p-value** | **Fit statistics^2^** |
| --- | --- | --- | --- | --- | --- | --- |
| **Emotional overeating and encouragement (ENC)** | | | | | | CFI = 0.944  RMSEA = 0.072 |
| Coupling parameter EOE to ∆ENC | γ1 | -0.06 | -0.11 | -0.01 | 0.024 |  |
| Coupling parameter ENC to ∆EOE | γ2 | 0.001 | -0.05 | 0.05 | 0.967 |  |
| Self-feedback parameter EOE | β1 | -0.76 | -0.70 | -0.81 | <0.001 |  |
| Self-feedback parameter ENC | β2 | -0.58 | -0.53 | -0.63 | <0.001 |  |
| Covariance between EOE & ENC at 15 months | Ф | 0.01 | -0.002 | 0.03 | 0.098 |  |
| Correlated change | ρ | -0.01 | -0.02 | 0.01 | 0.215 |  |
| **Food responsiveness and encouragement (ENC)** | | | | | | CFI = 0.964  RMSEA = 0.067 |
| Coupling parameter FR to ∆ENC | γ1 | 0.01 | -0.03 | 0.04 | 0.802 |  |
| Coupling parameter ENC to ∆FR | γ2 | -0.02 | -0.09 | 0.04 | 0.500 |  |
| Self-feedback parameter FR | β1 | -0.59 | -0.53 | -0.64 | <0.001 |  |
| Self-feedback parameter ENC | β2 | -0.58 | -0.53 | -0.63 | <0.001 |  |
| Covariance between FR and ENC at 15 months | Ф | 0.01 | -0.01 | 0.03 | 0.395 |  |
| Correlated change | ρ | -0.01 | -0.03 | 0.01 | 0.421 |  |
| **Enjoyment of food and encouragement (ENC)** | | | | | | CFI = 0.968  RMSEA = 0.062 |
| Coupling parameter EF to ∆ENC | γ1 | **0.07** | **0.03** | **0.11** | **0.001** |  |
| Coupling parameter ENC to ∆EF | γ2 | **0.06** | **0.004** | **0.11** | **0.006** |  |
| Self-feedback parameter EF | β1 | -0.53 | -0.47 | -0.59 | <0.001 |  |
| Self-feedback parameter ENC | β2 | -0.59 | -0.54 | -0.64 | <0.001 |  |
| Covariance between EF and ENC at 15 months | Ф | 0.03 | 0.02 | 0.05 | <0.001 |  |
| Correlated change | ρ | 0.02 | 0.01 | 0.04 | 0.010 |  |
| **Satiety responsiveness and encouragement (ENC)** | | | | | | CFI = 0.950  RMSEA = 0.077 |
| Coupling parameter SR to ∆ENC | γ1 | -0.01 | -0.06 | 0.03 | 0.510 |  |
| Coupling parameter ENC to ∆SR | γ2 | 0.02 | -0.03 | 0.07 | 0.358 |  |
| Self-feedback parameter SR | β1 | -0.59 | -0.54 | -0.64 | <0.001 |  |
| Self-feedback parameter ENC | β2 | -0.58 | -0.53 | -0.63 | <0.001 |  |
| Covariance between SR and ENC at 15 months | Ф | -0.004 | -0.02 | 0.01 | 0.683 |  |
| Correlated change | ρ | 0.01 | -0.01 | 0.02 | 0.365 |  |
| **Slowness in eating and encouragement (ENC)** | | | | | | CFI = 0.944  RMSEA = 0.070 |
| Coupling parameter SE to ∆ENC | γ1 | 0.01 | -0.03 | 0.05 | 0.740 |  |
| Coupling parameter ENC to ∆SE | γ2 | 0.05 | -0.01 | 0.10 | 0.121 |  |
| Self-feedback parameter SE | β1 | -0.67 | -0.62 | -0.73 | <0.001 |  |
| Self-feedback parameter ENC | β2 | -0.58 | -0.53 | -0.63 | <0.001 |  |
| Covariance between SE and ENC at 15 months | Ф | 0.00 | -0.02 | 0.02 | 0.905 |  |
| Correlated change | ρ | 0.02 | 0.00 | 0.03 | 0.087 |  |
| **Food fussiness and encouragement** | | | | | | CFI = 0.964  RMSEA = 0.064 |
| Coupling parameter FF to ∆ENC | γ1 | -0.04 | -0.08 | -0.01 | 0.022 |  |
| Coupling parameter ENC to ∆FF | γ2 | -0.03 | -0.10 | 0.04 | 0.382 |  |
| Self-feedback parameter FF | β1 | -0.50 | -0.45 | -0.56 | <0.001 |  |
| Self-feedback parameter ENC | β2 | -0.58 | -0.53 | -0.63 | <0.001 |  |
| Covariance between FF and ENC at 15 months | Ф | 0.01 | -0.02 | 0.02 | 0.627 |  |
| Correlated change | ρ | -0.01 | -0.02 | 0.01 | 0.546 |  |
| ^1^ Models adjusted for clustering within families and covariates; child age at measurements, SES, gestational age, child sex. Significant results for the parameters of interest in this study are shown in bold. ^2^Model fit indices were calculated, with cut-offs in parentheses indicating acceptable to good fit: Comparative Fit Index (CFI ≥ 0.90), Root Mean Square Error of Approximation (RMSEA ≤ 0.10). **Explanation of the parameters: Coupling parameters** **(γ1 & γ2)** – reflects the extent to which baseline levels in one domain (e.g. eating behaviour or parent feeding) predicts the rate or degree of change in the other domain (e.g. parent feeding or eating behaviour). A positive relationship for the **eating behaviour to parent feeding coupling parameter (γ1)** would indicate that higher eating behaviour scores at 15 months predicted greater increases in the parental feeding practice from 15 months to 5 years. A positive relationship for the **parent feeding to eating behaviour coupling parameter** **(γ2)** would indicate that higher parental feeding scores at 15 m predicted greater increases in the eating behaviour trait from 15m to 5 years.  **Self-feedback parameters (β1 and β2)** – reflects the extent to which baseline levels in one domain (e.g. eating behaviour or parent feeding) influences change in the same domain (e.g. eating behaviour or parental feeding). The self-feedback parameter is often negative reflecting regression towards the mean or ceiling effects and should not be overinterpreted.  **Covariance at 15 months (Φ)** – reflects the covariance between parental feeding practices and eating behaviour at 15 months.  **Correlated change (ρ)** - reflects the degree to which PFPs changes and eating behaviour changes co-occur after taking into account the coupling parameters. A positive relationship for correlated change indicates that over the 4-year period, greater increases in the eating behaviour trait were also associated with greater increases in the parental feeding practice. | | | | | | |

**Table S12.** Parameter estimates for bivariate latent change model^1^ between pressure to eat and six child eating behaviour traits using maximum likelihood with missing values (MLMV) n=3787.

| **Parameter** | | **Estimate** | **95% CI** | | **p-value** | **Fit statistics^2^** |
| --- | --- | --- | --- | --- | --- | --- |
| **Emotional overeating and pressure to eat (PRE)** | | | | | | CFI = 0.984  RMSEA = 0.037 |
| Coupling parameter EOE to ∆PRE | γ1 | 0.05 | -0.01 | 0.11 | 0.094 |  |
| Coupling parameter PRE to ∆EOE | γ2 | **0.07** | **0.03** | **0.11** | **0.001** |  |
| Self-feedback parameter EOE | β1 | -0.76 | -0.70 | -0.82 | <0.001 |  |
| Self-feedback parameter PRE | β2 | -0.59 | -0.54 | -0.64 | <0.001 |  |
| Covariance between EOE and PRE at 15 months | Ф | 0.02 | 0.004 | 0.04 | 0.017 |  |
| Correlated change | ρ | 0.01 | -0.01 | 0.03 | 0.206 |  |
| **Food responsiveness and pressure to eat (PRE)** | | | | | | CFI = 0.992  RMSEA = 0.031 |
| Coupling parameter FR to ∆PRE | γ1 | **0.07** | **0.03** | **0.11** | **0.001** |  |
| Coupling parameter PRE to ∆FR | γ2 | 0.05 | -0.00 | 0.10 | 0.057 |  |
| Self-feedback parameter FR | β1 | -0.59 | -0.53 | -0.64 | <0.001 |  |
| Self-feedback parameter PRE | β2 | -0.59 | -0.54 | -0.64 | <0.001 |  |
| Covariance between FR and PRE at 15 months | Ф | 0.01 | -0.02 | 0.04 | 0.418 |  |
| Correlated change | ρ | -0.04 | -0.07 | -0.02 | 0.001 |  |
| **Enjoyment of food and pressure to eat (PRE)** | | | | | | CFI = 0.972  RMSEA = 0.060 |
| Coupling parameter EF to ∆PRE | γ1 | -0.003 | -0.06 | 0.06 | 0.936 |  |
| Coupling parameter PRE to ∆EF | γ2 | -0.02 | -0.07 | 0.03 | 0.490 |  |
| Self-feedback parameter EF | β1 | -0.53 | -0.47 | -0.59 | <0.001 |  |
| Self-feedback parameter PRE | β2 | -0.59 | -0.54 | -0.64 | <0.001 |  |
| Covariance between EF and PRE at 15 months | Ф | -0.12 | -0.14 | -0.09 | <0.001 |  |
| Correlated change | ρ | -0.10 | -0.08 | -0.13 | <0.001 |  |
| **Satiety responsiveness and pressure to eat (PRE)** | | | | | | CFI = 0.980  RMSEA = 0.049 |
| Coupling parameter SR to ∆PRE | γ1 | -0.02 | -0.08 | 0.04 | 0.448 |  |
| Coupling parameter PRE to ∆SR | γ2 | 0.02 | -0.02 | 0.07 | 0.275 |  |
| Self-feedback parameter SR | β1 | -0.60 | -0.55 | -0.65 | <0.001 |  |
| Self-feedback parameter PRE | β2 | -0.59 | -0.54 | -0.64 | <0.001 |  |
| Covariance between SR and PRE at 15 months | Ф | 0.10 | 0.08 | 0.12 | <0.001 |  |
| Correlated change | ρ | 0.08 | 0.06 | 0.11 | <0.001 |  |
| **Slowness in eating and pressure to eat (PRE)** | | | | | | CFI = 0.986  RMSEA = 0.039 |
| Coupling parameter SE to ∆PRE | γ1 | -0.004 | -0.06 | 0.05 | 0.891 |  |
| Coupling parameter PRE to ∆SE | γ2 | **0.11** | **0.05** | **0.16** | **<0.001** |  |
| Self-feedback parameter SE | β1 | -0.69 | -0.64 | -0.75 | <0.001 |  |
| Self-feedback parameter PRE | β2 | -0.59 | -0.54 | -0.64 | <0.001 |  |
| Covariance between SE and PRE at 15 months | Ф | 0.09 | 0.07 | 0.11 | <0.001 |  |
| Correlated change | ρ | 0.14 | 0.12 | 0.17 | <0.001 |  |
| **Food fussiness and pressure to eat (PRE)** | | | | | | CFI = 0.995  RMSEA = 0.024 |
| Coupling parameter FF to ∆PRE | γ1 | 0.03 | -0.03 | 0.08 | 0.289 |  |
| Coupling parameter PRE to ∆FF | γ2 | 0.01 | -0.05 | 0.07 | 0.824 |  |
| Self-feedback parameter FF | β1 | -0.51 | -0.45 | -0.57 | <0.001 |  |
| Self-feedback parameter PRE | β2 | -0.60 | -0.55 | -0.65 | <0.001 |  |
| Covariance between FF and PRE at 15 months | Ф | 0.13 | 0.11 | 0.16 | <0.001 |  |
| Correlated change | ρ | 0.10 | 0.07 | 0.12 | <0.001 |  |
| ^1^ Models adjusted for clustering within families and covariates; child age at measurements, sex, SES, gestational age. Significant results for the parameters of interest in this study are shown in bold. ^2^Model fit indices were calculated, with cut-offs in parentheses indicating acceptable to good fit: Comparative Fit Index (CFI ≥ 0.90), Root Mean Square Error of Approximation (RMSEA ≤ 0.10). **Explanation of the parameters:**   - **Coupling parameters** **(γ1 & γ2)** – reflects the extent to which baseline levels in one domain (e.g. eating behaviour or parent feeding) predicts the rate or degree of change in the other domain (e.g. parent feeding or eating behaviour). A positive relationship for the **eating behaviour to parent feeding coupling parameter (γ1)** would indicate that higher eating behaviour scores at 15 months predicted greater increases in the parental feeding practice from 15 months to 5 years. A positive relationship for the **parent feeding to eating behaviour coupling parameter** **(γ2)** would indicate that higher parental feeding scores at 15 months predicted greater increases in the eating behaviour trait from 15 m to 5 years. - **Self-feedback parameters (β1 and β2)** – reflects the extent to which baseline levels in one domain (e.g. eating behaviour or parent feeding) influences change in the same domain (e.g. eating behaviour or parental feeding). The self-feedback parameter is often negative which reflects regression towards the mean or ceiling effects and should not be overinterpreted. - **Covariance at 15 months (Φ)** – reflects the covariance between parental feeding practices and eating behaviour at 15 months. - **Correlated change (ρ)** - reflects the degree to which PFPs changes and eating behaviour changes co-occur after taking into account the coupling parameters. A positive relationship for correlated change indicates that over the 4-year period, greater increases in the eating behaviour trait were also associated with greater increases in the parental feeding practice. | | | | | | |

**Table S13.** Parameter estimates for bivariate latent change model between parent control and six child eating behaviour traits using maximum likelihood with missing values (MLMV) n=3787.

| **Parameter** | | **Estimate** | **95% CI** | | **p-value** | **Fit statistics** |
| --- | --- | --- | --- | --- | --- | --- |
| **Emotional overeating and control (CON)** | | | | | | CFI = 0.908  RMSEA = 0.091 |
| Coupling parameter EOE to ∆CON | γ1 | 0.02 | -0.03 | 0.06 | 0.439 |  |
| Coupling parameter CON to ∆EOE | γ2 | **-0.10** | **-0.03** | **-0.17** | **0.006** |  |
| Self-feedback parameter EOE | β1 | -0.77 | -0.71 | -0.83 | <0.001 |  |
| Self-feedback parameter CON | β2 | -0.55 | -0.49 | -0.60 | <0.001 |  |
| Covariance between EOE and CON at 15 months | Ф | -0.04 | -0.02 | -0.05 | <0.001 |  |
| Correlated change | ρ | -0.02 | -0.01 | -0.03 | 0.001 |  |
| **Food responsiveness and control (CON)** | | | | | | CFI = 0.945  RMSEA = 0.080 |
| Coupling parameter FR to ∆CON | γ1 | 0.01 | -0.02 | 0.04 | 0.637 |  |
| Coupling parameter CON to ∆FR | γ2 | -0.05 | -0.13 | 0.03 | 0.239 |  |
| Self-feedback parameter FR | β1 | -0.59 | -0.53 | -0.64 | <0.001 |  |
| Self-feedback parameter CON | β2 | -0.55 | -0.49 | -0.60 | <0.001 |  |
| Covariance between FR and CON at 15 months | Ф | -0.03 | -0.05 | -0.02 | <0.001 |  |
| Correlated change | ρ | -0.02 | -0.03 | -0.003 | 0.019 |  |
| **Enjoyment of food and control (CON)** | | | | | | CFI = 0.951  RMSEA = 0.075 |
| Coupling parameter EF to ∆CON | γ1 | 0.04 | 0.00 | 0.08 | 0.048 |  |
| Coupling parameter CON to ∆EF | γ2 | 0.05 | -0.03 | 0.12 | 0.234 |  |
| Self-feedback parameter EF | β1 | -0.53 | -0.48 | -0.59 | <0.001 |  |
| Self-feedback parameter CON | β2 | -0.56 | -0.50 | -0.62 | <0.001 |  |
| Covariance between EF and CON at 15 months | Ф | 0.04 | 0.03 | 0.06 | <0.001 |  |
| Correlated change | ρ | 0.03 | 0.02 | 0.04 | <0.001 |  |
| **Satiety responsiveness and control (CON)** | | | | | | CFI = 0.919  RMSEA = 0.096 |
| Coupling parameter SR to ∆CON | γ1 | -0.02 | -0.06 | 0.02 | 0.424 |  |
| Coupling parameter CON to ∆SR | γ2 | -0.01 | -0.08 | 0.05 | 0.718 |  |
| Self-feedback parameter SR | β1 | -0.59 | -0.54 | -0.64 | <0.001 |  |
| Self-feedback parameter CON | β2 | -0.55 | -0.50 | -0.61 | <0.001 |  |
| Covariance between SR and CON at 15 months | Ф | -0.03 | -0.02 | -0.05 | <0.001 |  |
| Correlated change | ρ | -0.02 | -0.04 | -0.01 | <0.001 |  |
| **Slowness in eating and control (CON)** | | | | | | CFI = 0.908  RMSEA = 0.087 |
| Coupling parameter SE to ∆CON | γ1 | -0.02 | -0.06 | 0.01 | 0.195 |  |
| Coupling parameter CON to ∆SE | γ2 | 0.002 | -0.08 | 0.08 | 0.966 |  |
| Self-feedback parameter SE | β1 | -0.67 | -0.62 | -0.73 | <0.001 |  |
| Self-feedback parameter CON | β2 | -0.55 | -0.50 | -0.61 | <0.001 |  |
| Covariance between SE and CON at 15 months | Ф | -0.04 | -0.05 | -0.02 | <0.001 |  |
| Correlated change | ρ | -0.01 | -0.02 | 0.01 | 0.349 |  |
| **Food fussiness and Control** | | | | | | CFI = 0.946  RMSEA = 0.078 |
| Coupling parameter FF to ∆CON | γ1 | -0.02 | -0.05 | 0.02 | 0.292 |  |
| Coupling parameter CON to ∆FF | γ2 | 0.01 | -0.08 | 0.11 | 0.752 |  |
| Self-feedback parameter FF | β1 | -0.50 | -0.45 | -0.56 | <0.001 |  |
| Self-feedback parameter CON | β2 | -0.55 | -0.50 | -0.61 | <0.001 |  |
| Covariance between FF and CON at 15 months | Ф | -0.04 | -0.02 | -0.05 | <0.001 |  |
| Correlated change | ρ | -0.04 | -0.02 | -0.05 | <0.001 |  |
| ^1^ Models adjusted for clustering within families and covariates; child age at measurements, SES, gestational age, child sex. Significant results for the parameters of interest in this study are shown in bold. ^2^Model fit indices were calculated, with cut-offs in parentheses indicating acceptable to good fit: Comparative Fit Index (CFI ≥ 0.90), Root Mean Square Error of Approximation (RMSEA ≤ 0.10). **Explanation of the parameters:**   - **Coupling parameters** **(γ1 & γ2)** – reflects the extent to which baseline levels in one domain (e.g. eating behaviour or parent feeding) predicts the rate or degree of change in the other domain (e.g. parent feeding or eating behaviour). A positive relationship for the **eating behaviour to parent feeding coupling parameter (γ1)** would indicate that higher eating behaviour scores at 15 m predicted greater increases in the parental feeding practice from 15 months to 5 years. A positive relationship for the **parent feeding to eating behaviour coupling parameter** **(γ2)** would indicate that higher parental feeding scores at 15 months predicted greater increases in the eating behaviour trait from 15 months to 5 years. - **Self-feedback parameters (β1 and β2)** – reflects the extent to which baseline levels in one domain (e.g. eating behaviour or parent feeding) influences change in the same domain (e.g. eating behaviour or parental feeding). The self-feedback parameter is often negative which reflects regression towards the mean or ceiling effects and should not be overinterpreted. - **Covariance at 15 months (Φ)** – reflects the covariance between parental feeding practices and eating behaviour at 15 months. - **Correlated change (ρ)** - reflects the degree to which PFPs changes and eating behaviour changes co-occur after taking into account the coupling parameters. A positive relationship for correlated change indicates that over the 4-year period, greater increases in the eating behaviour trait were also associated with greater increases in the parental feeding practice. | | | | | | |

**Table S14.** Parameter estimates for bivariate latent change model between covert restriction and six child eating behaviour traits using maximum likelihood with missing values (MLMV) n=3787.

| **Parameter** | | **Estimate** | **95% CI** | | **p-value** | **Fit statistics** |
| --- | --- | --- | --- | --- | --- | --- |
| **Emotional overeating and Covert restriction (COV)** | | | | | | CFI =0.993  RMSEA = 0.033 |
| Coupling parameter EOE to ∆COV | γ1 | 0.03 | -0.03 | 0.09 | 0.370 |  |
| Coupling parameter COV to ∆EOE | γ2 | -0.01 | -0.05 | 0.02 | 0.418 |  |
| Self-feedback parameter EOE | β1 | -0.76 | -0.70 | -0.81 | <0.001 |  |
| Self-feedback parameter COV | β2 | -0.47 | -0.42 | -0.51 | <0.001 |  |
| Covariance between EOE and COV at 15 months | Ф | 0.02 | -0.00 | 0.05 | 0.052 |  |
| Correlated change | ρ | -0.001 | -0.02 | 0.02 | 0.893 |  |
| **Food responsiveness and Covert restriction (COV)** | | | | | | CFI =0.997  RMSEA = 0.022 |
| Coupling parameter FR to ∆COV | γ1 | -0.002 | -0.05 | 0.06 | 0.940 |  |
| Coupling parameter COV to ∆FR | γ2 | -0.01 | -0.05 | 0.04 | 0.766 |  |
| Self-feedback parameter FR | β1 | -0.59 | -0.53 | -0.64 | <0.001 |  |
| Self-feedback parameter COV | β2 | -0.46 | -0.42 | -0.51 | <0.001 |  |
| Covariance between FR and COV at 15 months | Ф | -0.02 | -0.05 | 0.01 | 0.185 |  |
| Correlated change | ρ | -0.02 | -0.04 | 0.01 | 0.232 |  |
| **Enjoyment of food and Covert restriction (COV)** | | | | | | CFI =0.991  RMSEA = 0.040 |
| Coupling parameter EF to ∆COV | γ1 | -0.002 | -0.06 | 0.06 | 0.960 |  |
| Coupling parameter COV to ∆EF | γ2 | 0.05 | 0.01 | 0.08 | 0.018 |  |
| Self-feedback parameter EF | β1 | -0.53 | -0.48 | -0.59 | <0.001 |  |
| Self-feedback parameter COV | β2 | -0.46 | -0.42 | -0.51 | <0.001 |  |
| Covariance between EF and COV at 15 months | Ф | 0.02 | 0.00 | 0.05 | 0.061 |  |
| Correlated change | ρ | 0.02 | -0.01 | 0.04 | 0.169 |  |
| **Satiety responsiveness and Covert restriction (COV)** | | | | | | CFI =0.986  RMSEA = 0.049 |
| Coupling parameter SR to ∆COV | γ1 | -0.01 | -0.07 | 0.06 | 0.839 |  |
| Coupling parameter COV to ∆SR | γ2 | -0.02 | -0.05 | 0.02 | 0.277 |  |
| Self-feedback parameter SR | β1 | -0.59 | -0.54 | -0.64 | <0.001 |  |
| Self-feedback parameter COV | β2 | -0.46 | -0.42 | -0.51 | <0.001 |  |
| Covariance between SR and COV at 15 months | Ф | 0.02 | -0.004 | 0.05 | 0.095 |  |
| Correlated change | ρ | 0.003 | -0.02 | 0.02 | 0.755 |  |
| **Slowness in eating and Covert restriction (COV)** | | | | | | CFI =0.988  RMSEA = 0.042 |
| Coupling parameter SE to ∆COV | γ1 | -0.02 | -0.08 | 0.03 | 0.397 |  |
| Coupling parameter COV to ∆SE | γ2 | -0.05 | -0.01 | -0.09 | 0.019 |  |
| Self-feedback parameter SE | β1 | -0.67 | -0.61 | -0.73 | <0.001 |  |
| Self-feedback parameter COV | β2 | -0.46 | -0.42 | -0.51 | <0.001 |  |
| Covariance between SE and COV at 15 months | Ф | 0.02 | -0.00 | 0.05 | 0.076 |  |
| Correlated change | ρ | -0.01 | -0.04 | 0.01 | 0.323 |  |
| **Food fussiness and Covert restriction** | | | | | | CFI = 0.999  RMSEA = 0.011 |
| Coupling parameter FF to ∆COV | γ1 | -0.02 | -0.07 | 0.04 | 0.621 |  |
| Coupling parameter COV to ∆FF | γ2 | -0.03 | -0.08 | 0.02 | 0.199 |  |
| Self-feedback parameter FF | β1 | -0.51 | -0.45 | -0.56 | <0.001 |  |
| Self-feedback parameter COV | β2 | -0.46 | -0.42 | -0.51 | <0.001 |  |
| Covariance between FF and COV at 15 months | Ф | -0.03 | -0.05 | 0.00 | 0.061 |  |
| Correlated change | ρ | -0.01 | -0.04 | 0.02 | 0.421 |  |
| ^1^ Models adjusted for clustering within families and covariates; child age at measurements, SES, gestational age, child sex. Significant results for the parameters of interest in this study are shown in bold. ^2^Model fit indices were calculated, with cut-offs in parentheses indicating acceptable to good fit: Comparative Fit Index (CFI ≥ 0.90), Root Mean Square Error of Approximation (RMSEA ≤ 0.10). **Explanation of the parameters: Coupling parameters** **(γ1 & γ2)** – reflects the extent to which baseline levels in one domain (e.g. eating behaviour or parent feeding) predicts the rate or degree of change in the other domain (e.g. parent feeding or eating behaviour). A positive relationship for the **eating behaviour to parent feeding coupling parameter (γ1)** would indicate that higher eating behaviour scores at 15 months predicted greater increases in the parental feeding practice from 15 months to 5 years. A positive relationship for the **parent feeding to eating behaviour coupling parameter** **(γ2)** would indicate that higher parental feeding scores at 15 months predicted greater increases in the eating behaviour trait from 15 months to 5 years.   - **Self-feedback parameters (β1 and β2)** – reflects the extent to which baseline levels in one domain (e.g. eating behaviour or parent feeding) influences change in the same domain (e.g. eating behaviour or parental feeding). The self-feedback parameter is often negative which reflects regression towards the mean or ceiling effects and should not be overinterpreted. - **Covariance at 15 months (Φ)** – reflects the covariance between parental feeding practices and eating behaviour at 15 months. - **Correlated change (ρ)** - reflects the degree to which PFPs changes and eating behaviour changes co-occur after taking into account the coupling parameters. A positive relationship for correlated change indicates that over the 4-year period, greater increases in the eating behaviour trait were also associated with greater increases in the parental feeding practice. | | | | | | |

**Table S15.** Parameter estimates for bivariate latent change model between monitoring and six child eating behaviour traits using maximum likelihood with missing values (MLMV) n=3787.

| **Parameter** | | **Estimate** | **95% CI** | | **p-value** | **Fit statistics** |
| --- | --- | --- | --- | --- | --- | --- |
| **Emotional overeating and monitoring (MON)** | | | | | | CFI = 0.990  RMSEA = 0.032 |
| Coupling parameter EOE to ∆MON | γ1 | 0.02 | -0.06 | 0.10 | 0.691 |  |
| Coupling parameter MON to ∆EOE | γ2 | 0.01 | -0.02 | 0.04 | 0.469 |  |
| Self-feedback parameter EOE | β1 | -0.76 | -0.70 | -0.81 | <0.001 |  |
| Self-feedback parameter MON | β2 | -0.55 | -0.49 | -0.61 | <0.001 |  |
| Covariance between EOE and MON at 15 months | Ф | -0.02 | -0.04 | 0.01 | 0.215 |  |
| Correlated change | ρ | -0.01 | -0.03 | 0.02 | 0.619 |  |
| **Food responsiveness and monitoring (MON)** | | | | | | CFI = 0.997  RMSEA = 0.020 |
| Coupling parameter FR to ∆MON | γ1 | 0.03 | -0.04 | 0.09 | 0.406 |  |
| Coupling parameter MON to ∆FR | γ2 | 0.02 | -0.02 | 0.06 | 0.298 |  |
| Self-feedback parameter FR | β1 | -0.59 | -0.53 | -0.64 | <0.001 |  |
| Self-feedback parameter MON | β2 | -0.55 | -0.50 | -0.61 | <0.001 |  |
| Covariance between FR and MON at 15 months | Ф | -0.02 | -0.06 | 0.01 | 0.135 |  |
| Correlated change | ρ | -0.01 | -0.04 | 0.02 | 0.500 |  |
| **Enjoyment of food and monitoring (MON)** | | | | | | CFI = 0.988  RMSEA = 0.039 |
| Coupling parameter EF to ∆MON | γ1 | 0.06 | -0.01 | 0.13 | 0.114 |  |
| Coupling parameter MON to ∆EF | γ2 | **0.05** | **0.02** | **0.09** | **0.005** |  |
| Self-feedback parameter EF | β1 | -0.54 | -0.48 | -0.59 | <0.001 |  |
| Self-feedback parameter MON | β2 | -0.56 | -0.50 | -0.61 | <0.001 |  |
| Covariance between EF and MON at 15 months | Ф | 0.06 | 0.03 | 0.09 | <0.001 |  |
| Correlated change | ρ | 0.05 | 0.02 | 0.07 | 0.001 |  |
| **Satiety responsiveness and monitoring (MON)** | | | | | | CFI = 0.980  RMSEA = 0.051 |
| Coupling parameter SR to ∆MON | γ1 | -0.03 | -0.11 | 0.04 | 0.385 |  |
| Coupling parameter MON to ∆SR | γ2 | -0.02 | -0.05 | 0.01 | 0.217 |  |
| Self-feedback parameter SR | β1 | -0.59 | -0.54 | -0.64 | <0.001 |  |
| Self-feedback parameter MON | β2 | -0.55 | -0.50 | -0.61 | <0.001 |  |
| Covariance between SR and MON at 15 months | Ф | -0.02 | -0.04 | 0.01 | 0.264 |  |
| Correlated change | ρ | -0.03 | -0.05 | 0.00 | 0.043 |  |
| **Slowness in eating and monitoring (MON)** | | | | | | CFI = 0.982  RMSEA = 0.042 |
| Coupling parameter SE to ∆MON | γ1 | -0.06 | -0.13 | 0.01 | 0.084 |  |
| Coupling parameter MON to ∆SE | γ2 | -0.01 | -0.04 | 0.03 | 0.784 |  |
| Self-feedback parameter SE | β1 | -0.67 | -0.62 | -0.73 | <0.001 |  |
| Self-feedback parameter MON | β2 | -0.55 | -0.50 | -0.61 | <0.001 |  |
| Covariance between SE and MON at 15 months | Ф | -0.02 | -0.04 | 0.01 | 0.205 |  |
| Correlated change | ρ | -0.01 | -0.04 | 0.02 | 0.419 |  |
| Food fussiness and monitoring | | | | | | CFI = 0.999  RMSEA = 0.013 |
| Coupling parameter FF to ∆MON | γ1 | -0.01 | -0.08 | 0.06 | 0.699 |  |
| Coupling parameter MON to ∆FF | γ2 | -0.02 | -0.07 | 0.02 | 0.311 |  |
| Self-feedback parameter FF | β1 | -0.51 | -0.45 | -0.57 | <0.001 |  |
| Self-feedback parameter MON | β2 | -0.55 | -0.50 | -0.61 | <0.001 |  |
| Covariance between FF and MON at 15 months | Ф | -0.08 | -0.05 | -0.11 | <0.001 |  |
| Correlated change | ρ | -0.02 | -0.05 | 0.02 | 0.298 |  |
| ^1^ Models adjusted for clustering within families and covariates; child age at measurements, sex, SES, gestational age. Significant results for the parameters of interest in this study are shown in bold. ^2^Model fit indices were calculated, with cut-offs in parentheses indicating acceptable to good fit: Comparative Fit Index (CFI ≥0.90), Root Mean Square Error of Approximation (RMSEA ≤ 0.10). **Explanation of the parameters:**   - **Coupling parameters** **(γ1 & γ2)** – reflects the extent to which baseline levels in one domain (e.g. eating behaviour or parent feeding) predicts the rate or degree of change in the other domain (e.g. parent feeding or eating behaviour). A positive relationship for the **eating behaviour to parent feeding coupling parameter (γ1)** would indicate that higher eating behaviour scores at 15 months predicted greater increases in the parental feeding practice from 15 months to 5 years. A positive relationship for the **parent feeding to eating behaviour coupling parameter** **(γ2)** would indicate that higher parental feeding scores at 15 months predicted greater increases in the eating behaviour trait from 15 m to 5 years. - **Self-feedback parameters (β1 and β2)** – reflects the extent to which baseline levels in one domain (e.g. eating behaviour or parent feeding) influences change in the same domain (e.g. eating behaviour or parental feeding). The self-feedback parameter is often negative which reflects regression towards the mean or ceiling effects and should not be overinterpreted. - **Covariance at 15 months (Φ)** – reflects the covariance between parental feeding practices and eating behaviour at 15 m. - **Correlated change (ρ)** - reflects the degree to which PFPs changes and eating behaviour changes co-occur after taking into account the coupling parameters. A positive relationship for correlated change indicates that over the 4-year period, greater increases in the eating behaviour trait were also associated with greater increases in the parental feeding practice. | | | | | | |
